# Synergistic Liposomal Delivery of Ibrexafungerp Citrate and Marine-Sourced Silver Nanoparticles for Effective Management of Vulvovaginal Candidiasis

**DOI:** 10.3390/jfb17060290

**Published:** 2026-06-09

**Authors:** Pottabathula Shyam Sundar, Uday Kumar S. Patil, Thombre Pooja Sarjerao, Somnath D. Bhinge, Sunil T. Galatage, Unnam Sambamoorthy, Rahul J. Kadam, Viswas Raja Solomon, Arehalli S. Manjappa

**Affiliations:** 1 Department of Pharmaceutical Chemistry, Vasantidevi Patil Institute of Pharmacy, Kodoli, Panahala, Kolhapur 416114, Maharashtra, India; shyam.pottabathula@yspm.in (P.S.S.); rahul.kadam@yspm.in (R.J.K.); 2 Department of Pharmaceutics, Bharati Vidyapeeth College of Pharmacy, Kolhapur 416013, Maharashtra, India; udaykumar.patil@bharatividyapeeth.edu; 3 Department of Pharmaceutical Quality Assurance, Vasantidevi Patil Institute of Pharmacy, Kodoli, Panahala, Kolhapur 416114, Maharashtra, India; poojathombre2001@gmail.com; 4 Krishna Institute of Pharmacy, Krishna Vishwa Vidyapeeth (Deemed To Be University), Karad 415539, Maharashtra, India; somu1245@gmail.com; 5 Department of Pharmaceutics, Vasantidevi Patil Institute of Pharmacy, Kodoli, Panhala, Kolhapur 416114, Maharashtra, India; 6 Department of Pharmaceutics, Sree Dattha Institute of Pharmacy, Sheriguda, Ibrahimpatnam, Rangareddy, Hyderabad 501510, Telangana, India; unnammoorthy@sreedattha.ac.in; 7 Medicinal Chemistry Research Laboratory, MNR College of Pharmacy, Sangareddy 502294, Telangana, India; vrajasolomon@gmail.com; 8 Department of Chemistry, University of Saskatchewan, Saskatoon, SK S7N 5A2, Canada

**Keywords:** vulvovaginal candidiasis, *Ascophyllum nodosum*, ibrexafungerp citrate, silver nanoparticle, green synthesis, liposomal delivery

## Abstract

Background: Increasing antifungal resistance, poor mucosal retention, and systemic side effects limit the effectiveness of currently available drugs. This study explores a novel topical nanotherapeutic approach for the targeted treatment of vulvovaginal candidiasis (VVC), employing green-synthesized silver nanoparticles (AgNPs) derived from *Ascophyllum nodosum* (AN) and incorporating ibrexafungerp citrate (IBC) into a liposomal formulation. Methods: AgNPs were biosynthesized using AN extract and characterized. Liposomes were prepared by thin-film hydration, and optimised using Central Composite design and characterized and optimized. Optimised liposomes, co-loaded with IBC and AN-AgNPs, were incorporated into a Carbopol-CMC-based topical gel. Results: FTIR shifts in the –OH (3332.31 cm^−1^) and carbonyl (1636.87 cm^−1^) bands with reduced intensity confirmed their involvement in Ag^+^ reduction and nanoparticle surface coordination, while the persistence of the 1015 cm^−1^ band indicated the role of polysaccharides in capping and stabilizing the AN-AgNP. Characterization of the optimized liposomes (IBCL-11) revealed a particle size of 127.2 nm, a zeta potential of −43.8 mV, and a polydispersity index (PDI) of 0.35. Transmission Electron Microscopy (TEM) confirmed the presence of intact, spherical vesicles, while Differential Scanning Calorimetry (DSC) and X-ray diffraction (XRD) validated the molecular dispersion and amorphous characteristics of the films. In vitro evaluations of the IBC liposomal gel demonstrated a sustained drug release of 72.6% over 24 h, alongside enhanced drug penetration across all skin layers. Antifungal assays highlighted the formulation’s potent efficacy, yielding Minimum Inhibitory Concentration (MIC) and Minimum Fungicidal Concentration (MFC) values below 1 µg/mL. Furthermore, the treatments exhibited strong anti-biofilm properties; at MIC and MBC levels, AN-AgNPs achieved biofilm reductions of 45.27 ± 3.16% and 27.62 ± 2.13%, respectively, whereas IBCL-11 produced reductions of 34.25 ± 2.43% and 16.28 ± 1.72%. Conclusion: Ultimately, this study successfully developed an eco-friendly liposomal formulation co-loaded with AN-AgNPs and IBC, offering a promising and targeted therapeutic approach for the treatment of vulvovaginal candidiasis.

## 1. Introduction

Vulvovaginal candidiasis (VVC) is one of the most prevalent mucosal fungal infections affecting women globally, with approximately 75% experiencing at least one episode during their lifetime and 8–10% suffering from recurrent infections [1]. The condition is primarily caused by *Candida albicans*, although non-*albicans* species such as *Candida glabrata* are increasingly implicated and present significant therapeutic challenges due to intrinsic and acquired resistance to conventional antifungal agents [2]. Current treatment strategies, predominantly based on azole antifungals, are often limited by poor drug retention at the site of infection, incomplete eradication of biofilms, systemic side effects, and high recurrence rates [3,4]. These limitations underscore the urgent need for alternative therapeutic approaches that can provide localized, sustained, and more effective antifungal action. In recent years, nanotechnology-based drug delivery systems have emerged as promising platforms to overcome these challenges by enhancing drug solubility, stability, and site-specific delivery [5]. Among these, liposomal systems have been extensively investigated due to their biocompatibility, ability to encapsulate both hydrophilic and lipophilic drugs, and capacity to improve mucosal permeation and retention [6,7]. Several studies have reported liposomal formulations of antifungal agents such as amphotericin B, fluconazole, and clotrimazole for vaginal delivery, demonstrating improved therapeutic outcomes and reduced toxicity [8,9]. However, despite these advances, there remains a critical gap in the development of liposomal systems for the localized delivery of newer antifungal agents such as ibrexafungerp, particularly in combination with multifunctional nanomaterials to address drug resistance and biofilm-associated infections. Silver nanoparticles (AgNPs) have been widely explored for their broad-spectrum antimicrobial and antibiofilm properties, including activity against drug-resistant *Candida* species [10,11]. While chemically synthesized AgNPs are well documented, concerns regarding toxicity and environmental impact have driven interest toward green synthesis approaches [12]. Plant and marine-derived AgNPs have demonstrated enhanced biological activity due to surface functionalization with bioactive phytochemicals [13]. Notably, marine macroalgae such as *Ascophyllum nodosum* (AN) are rich in sulfated polysaccharides (fucoidans) and polyphenols (phlorotannins), which possess intrinsic antifungal, antioxidant, and membrane-disrupting properties [14]. Recent studies have highlighted the synergistic antimicrobial potential of such biofunctionalized AgNPs; however, their integration into advanced vesicular drug delivery systems for mucosal fungal infections remains largely unexplored [15]. Ibrexafungerp citrate (IBC), a first-in-class triterpenoid glucan synthase inhibitor, has demonstrated potent fungicidal activity against both azole-sensitive and resistant *Candida* strains, including *C. glabrata* [16]. Although approved for oral use, its systemic administration may be associated with suboptimal bioavailability and off-target effects [17]. To date, there is a notable lack of studies investigating the topical or vaginal delivery of ibrexafungerp using nanocarrier systems, particularly those designed to enhance mucosal retention and synergistic antifungal efficacy. Therefore, the present study addresses this unmet need by developing a novel liposomal gel system co-loaded with ibrexafungerp and green-synthesized AgNPs derived from *Ascophyllum nodosum*. This approach is designed to combine (i) the potent fungicidal activity of ibrexafungerp, (ii) the intrinsic antifungal and biofilm-disrupting properties of AN-mediated AgNPs, and (iii) the mucosal targeting and sustained release capabilities of liposomal carriers. The formulation was further incorporated into a Carbopol 934-based gel to enhance vaginal retention and stability. A Quality by Design (QbD) framework was employed to optimize formulation parameters, followed by comprehensive physicochemical characterization and biological evaluation. This study thus provides a previously unexplored, synergistic, and targeted nano-enabled strategy for the topical management of VVC, particularly relevant in the context of rising antifungal resistance and recurrent infections.

## 2. Materials and Methods

### 2.1. Materials

AN extract was procured from Aseschem Lab (Jodhpur, Rajasthan, India). IBC was obtained from Yarro Chem (Mumbai, Maharashtra, India). Analytical-grade methanol, chloroform, cholesterol, and Carbopol 934 were purchased from Loba Chemie (Mumbai, Maharashtra, India). Silver nitrate (AgNO_3_), sodium hydroxide (NaOH), triethanolamine (TEA), carboxymethyl cellulose, and methyl paraben were sourced from Molychem (Mumbai, Maharashtra, India), and hydrogenated soy phosphatidylcholine (HSPC) from Lipoid (Ludwigshafen, Rhineland-Palatinate, Germany). Deionised water was supplied by Unichem Lab (Kolhapur, Maharashtra, India). All chemicals were of analytical quality and utilized without additional purification. Design-Expert^®^ software (Version 23, Stat-Ease Inc., Minneapolis, MI, USA) was used for the design of experiments (DOE), optimization, and statistical analysis.

### 2.2. Extraction of Plant Material

Using 250 mL of methanol as the solvent and 25 g of finely ground material, a Soxhlet extraction was performed. To ensure optimal recovery of phytoconstituents, the extraction was conducted for a predetermined period. The methanolic solution was concentrated at 40 °C under reduced pressure using a rotary evaporator to preserve the chemical integrity of the bioactive components following extraction. A concentrated crude extract was obtained by drying the extract at 40 °C for 1 h to further evaporate residual solvent. The extract was weighed and stored at 4 °C in sealed containers to avert degradation and maintain stability for subsequent analysis [18,19].

### 2.3. Preparation of AN-AgNPs

10 mL of aqueous AN extract was incrementally added to 90 mL of a 1 mM AgNO_3_ solution (MW 169.87 g/mol), and the mixture was agitated at ambient temperature. The pH of the reaction mixture was adjusted to 8 by incorporating a 1% *w*/*v* NaOH (MW 40 g/mol) solution, followed by a 24 h incubation in the dark at ambient temperature for bio-reduction. Upon incubation, the reaction mixture transforms from light brown to dark brown, signifying the synthesis of AgNPs. The synthesized AgNPs were isolated from the reaction mixture using centrifugation at 10,000 rpm for 20 min at 40 °C. The particle was rinsed and resuspended in Milli-Q water, followed by an additional centrifugation step. This purification procedure was conducted thrice to eliminate any remaining extract [14]. The purified AgNPs, acquired as a pellet, were dried at 60 °C to form a fine powder for subsequent characterization.

### 2.4. Characterisation of AN-AgNPs

The synthesis of AN-AgNPs was visually indicated by a color shift from light to dark brown and confirmed via UV-Vis spectrophotometry (SHIMADZU UV-1900, 200–800 nm). Reaction kinetics were monitored over 24 h by tracking the surface plasmon resonance (SPR) peak at 416 nm [20]. To confirm synthesis and stabilization, FTIR spectra (Bruker Alpha-II, KBr pellet method, Karlsruhe, Germany) of the AN extract, AgNO_3_, physical mixture, and AN-AgNPs were recorded from 4000 to 400 cm^−1^ at a resolution of 4 cm^−1^ (32 scans per sample) [15]. Particle size, PDI, and zeta potential were measured in triplicate using a Malvern Zetasizer Nano ZS90 (Malvern, UK) at 25 °C (90° angle) after a 1:10 aqueous dilution. The thermal behavior of IBC, HSPC, and cholesterol was analyzed in triplicate using a Shimadzu DSC-60, heating 5 mg samples from 25 to 300 °C at 10 °C/min under nitrogen [21]. Morphology was evaluated in triplicate using high-resolution TEM (JEOL JEM-2100, Peabody, MA, USA). Ultrasonicated samples were deposited onto 300-mesh carbon-coated copper grids and stained with 1% uranyl acetate. Average particle size was calculated using ImageJ software (Version 1.53t) from at least 100 nanoparticles [22]. Elemental composition was determined using SEM-EDX (JEOL JSM-IT200) at 10 kV. To ensure sample homogeneity, spectra were recorded from three randomly selected areas of the dried dispersion on an aluminum stub [23].

### 2.5. Formulation, Optimisation and Characterisation of IBC-Liposomes

#### 2.5.1. Compatibility Studies

FTIR and DSC analyses were conducted to evaluate the physicochemical compatibility among IBC, AN-AgNPs, HSPC, cholesterol, optimised IBCL-11, and the excipients used in the liposomal formulation. The FTIR spectrophotometer was employed to obtain FTIR spectra of IBC, AN-AgNPs, HSPC, Cholesterol, and optimized IBCL-11, along with liposomal formulation excipients, throughout the 4000–650 cm^−1^ frequency range [24]. DSC thermograms (DSC-60, Shimadzu, Japan) of IBC, HSPC, cholesterol, and optimized IBCL-11, together with liposomal formulation excipients, were conducted to investigate alterations in their thermal properties [25].

#### 2.5.2. Formulation

IBC-loaded liposomes (IBCL) were synthesized using the thin-film hydration technique [26]. In summary, IBC (20 mg; MW 922.2 g/mol) and the liposomal constituents, specifically HSPC (30 mg; MW 790.1 g/mol) and cholesterol (15 mg; MW 386.6 g/mol), were accurately measured and dissolved in 15 mL of a methanol: chloroform combination (1:2, *v*/*v*) via bath sonication. Organic solvents were subsequently evaporated at 65 ± 2 °C utilizing a rotary evaporator (BUCHI Rotavapor R-200, Mumbai, India), resulting in a thin lipid layer on the inner surface of the round-bottom flask. The lipid film was subsequently vacuum-dried overnight to eliminate any residual solvents. The dried lipid film was ultimately hydrated with 40 mL of sterile water for injection (SWI) at 70 ± 2 °C for 15 min with gentle agitation to produce multilamellar vesicles (MLVs). The MLVs were further exposed to probing sonication (LABMAN Pro-656, Chennai, India) to diminish vesicle size and generate small unilamellar vesicles (SUVs). To avert lipid breakdown and drug leakage due to heat created during the procedure, the sample vial was submerged in an ice-water bath to sustain a temperature below 10 °C. Probe sonication was conducted for five cycles, each lasting one minute, consisting of 12 s of sonication at 240 V and 0.6 A, followed by an 8 s interval [27].

#### 2.5.3. Optimisation

##### Experimental Design

A 3^2^ full factorial design has been employed for the systematic generation and optimization of IBC-Liposomes using Design Expert^®^ software (Version 7.0.0, Stat-Ease Inc., Minneapolis, MN, USA). Two independent variables, X_1_ (HSPC concentration) and X_2_ (cholesterol concentration), were examined at three levels (−1, 0, +1) to assess their impact on Y_1_ (particle size) and Y_2_ (EE). A total of 13 experimental runs were conducted according to the design (Table 1), and all formulations were assessed for particle size and EE. The responses have been adapted to multiple models (linear, quadratic, two-factor interaction, cubic). The optimal model was chosen based on one-way analysis of variance (ANOVA) results, taking into account R^2^, adjusted R^2^, predicted R^2^, and *p*-values, subsequently applying Dunnett’s multiple-comparison test. A *p*-value of less than 0.05 was deemed statistically significant. Response surface plots, contour plots, and perturbation graphs were created using GraphPad Prism 5.0 (GraphPad Software, La Jolla, CA, USA) to illustrate the impacts of the variables. The optimized formulation was determined using numerical and graphical optimization employing a desirability function, aiming for a low particle size and a maximum EE. An overlay plot highlighted the most optimal region, and the optimized formulation was generated to validate the model by comparing predicted and experimental responses.

### 2.6. Characterization of Optimized Liposomal Formulation

The particle size and zeta potential of the optimized IBCL-11 formulation were measured in triplicate using a Malvern Zetasizer at 25 ± 1 °C and 150 mV. Samples were diluted with double-distilled water prior to analysis, and results are reported as mean ± SD [28]. Entrapment efficiency (EE) was determined indirectly. The liposomal dispersion was centrifuged at 10,000 rpm for 1 h, and the unentrapped drug in the supernatant was diluted with methanol and quantified via UV-Vis spectrophotometry at 286 nm [29]. Morphology was evaluated using transmission electron microscopy (JEOL JEM 1400, Peabody, MA, USA). A 5 µL sample was placed on a 300-mesh carbon-coated copper grid, negatively stained with 1% (*w*/*v*) uranyl acetate for 3–5 min, air-dried, and imaged. The physical state and crystallinity of pure IBC and the optimized IBCL-11 formulation were analyzed using an X-ray diffractometer (Bruker D2 PHASER, Karlsruhe, Germany) to detect any phase transitions upon encapsulation [30].

### 2.7. Preparation of IBC-Liposomal Gel

Carbopol-934 (1.0 g) was dispersed in 50 mL of warm distilled water and allowed to hydrate for 2 h, then homogenised with a magnetic stirrer at 600 rpm. Separately, carboxymethyl cellulose (1.0 g) and methyl paraben (0.2 mL) were dissolved in 50 mL of warm distilled water under continuous stirring to form a viscous gel base. The two aqueous phases were combined with constant agitation. The pH of the dispersion was adjusted to neutrality by dropwise addition of TEA (q.s.). The optimised liposomal formulation (10 mL) was incorporated into the gel base, followed by the addition of propylene glycol (0.5 mL) as a permeation enhancer. The final mixture was agitated until a smooth, homogeneous gel was obtained [31].

### 2.8. Characterization of Liposomal Gel

To assess the drug content, 1 g of the optimized liposomal gel was weighed and dissolved in methanol. The mixture was then centrifuged, and the supernatant was filtered and analyzed by UV-spectrophotometry. The formulation’s physical properties were assessed by visual inspection for homogeneity and appearance. Furthermore, the pH was measured with a calibrated digital pH meter to evaluate its suitability for topical use. Rheological properties, particularly viscosity, were measured using a Brookfield viscometer (AMETEK, Berwyn, PA, USA) [26].

### 2.9. In Vitro Drug Release Study

The drug release profile of IBC has been analyzed at Simulated Vaginal Fluid (SVF), pH 4.2 via the dialysis bag method in a USP Type II (paddle) apparatus at 37 ± 2 °C. IBCL-11 dispersions, containing an equivalent of 5 mg of drug, have been placed in dialysis bags (LA390-10mt-HiMedia, Kennett Square, PA, USA), securely sealed, affixed to the paddle, and individually immersed in vessels containing 100 mL of Simulated Vaginal Fluid (SVF), pH 4.2, each supplemented with 5% (*v*/*v*) methanol. The media were continuously stirred at 100 rpm and maintained at 37 °C. At specified time intervals (0, 1, 2, 4, 6, 8, 10, 12, 24 h), 3 mL samples were extracted and analyzed using a UV-visible spectrophotometer at 286 nm. A fresh buffer has been incorporated into the withdrawn volume to maintain sink conditions. The percentage of drug released was computed and graphed as a function of time [32].

### 2.10. In Vitro Antifungal Activity

#### 2.10.1. Minimum Inhibitory Concentration (MIC)

The antifungal efficacy of the AN extract, IBC drug (reference), AN-AgNPs, and the liposomal formulation was assessed against two pathogenic Candida species: *Candida albicans* and *Candida glabrata*. The MIC was determined by serial dilution in Fluid Thioglycollate Medium. An initial stock solution was prepared by combining 20 µL of the test formulation with 380 µL of sterile thioglycollate broth. A dilution series was prepared using nine additional tubes, each pre-filled with 200 µL of sterile broth. A serial dilution was performed by transferring 200 µL from the initial stock tube into the first dilution tube. Subsequently, 200 µL was transferred sequentially from tube to tube to achieve a geometric dilution series, culminating in the ninth tube. In parallel, inoculum suspensions of *C. albicans* and *C. glabrata* were prepared by transferring 5 µL of the respective stock cultures into 2 mL of thioglycollate broth. From these standardized suspensions, 200 µL of inoculum was added to each tube containing the test formulations (AN extract, IBC, AN-AgNPs, and Liposomes). The tubes were incubated anaerobically at 37 °C for 48 to 72 h. After incubation, the tubes were visually examined for turbidity, indicating fungal growth. The MIC was defined as the lowest concentration of the formulation that completely inhibited visible growth of the Candida species [33].

#### 2.10.2. Minimum Fungicidal Concentration (MFC)

The MFC assay was performed to determine whether the test samples exhibited fungistatic or fungicidal activity against two pathogenic Candida species: *Candida albicans* and *Candida glabrata*. First, agar plates were prepared from samples (AN extract, IBC drug (reference), AN-AgNPs, Liposomes) taken from MIC tubes showing microbial sensitivity, and incubated for 24–48 h. The next day, colony counts were performed to assess fungal viability. In this context, an extract showing no colony formation on the agar was classified as fungicidal, indicating complete destruction of the organism. Conversely, any growth indicated a fungistatic effect, meaning the sample halted growth without killing the fungi [34].

#### 2.10.3. Time Kill Assay

The bactericidal kinetics of AN extract, IBC, AN-AgNPs and liposomes were evaluated using the microbroth dilution method. Bacterial suspensions of *C. albicans* were incubated with the test formulations at control, MIC, and MFC concentrations in sterile microcentrifuge tubes. For time kill assays, untreated bacterial cultures were used as growth controls, while cultures treated with PS extract alone served as formulation controls. Liposomal–treated cultures were compared against these controls under identical incubation conditions to assess time-dependent bactericidal activity. At predetermined time intervals (0, 4, 8, 12, 24, and 48 h), aliquots were withdrawn, serially diluted, and spread onto blood agar plates. After incubation at 37 °C for 48 h, colony-forming units (CFUs) were enumerated using a digital colony counter. The log_10_ CFU/mL values were plotted against time for constructing time-kill curves, providing insight into rate and extent of bacterial killing [35].

#### 2.10.4. Biofilm Reduction Crystal Violet Assay

Biofilm formation was assessed using the crystal violet staining method. Bacterial cultures were adjusted to 0.5 McFarland standard and inoculated into 96-well polystyrene microplates containing brain heart infusion (BHI) broth, followed by aerobic incubation at 37 °C for 72 h to allow biofilm development. After incubation, planktonic cells were removed, and wells were gently washed with phosphate-buffered saline (PBS). Preformed biofilms were then treated with AN extract, IBC, AN-AgNPs and liposomes at MIC and MFC concentrations, while untreated wells served as negative controls. Following treatment, biofilms were fixed with methanol, stained with 0.1% (*w*/*v*) crystal violet, and excess stain was removed by washing. The bound dye was solubilized using 95% ethanol, and absorbance was measured at 570 nm using a microplate reader to quantify biofilm biomass [36,37].

### 2.11. Ex Vivo Skin Permeation Study

To assess liposome penetration through the skin layers, fluorescence microscopy was used. Goat ear skin was uniformly treated (IAEC approval number: TKCP/22/01/23) with Rhodamine 6G (Rh6G) solution (control) and Rh6G-loaded liposomes, then mounted in a Franz diffusion cell. After 8 h, the skin was removed, thoroughly rinsed with water to remove excess formulation, and the treated sections were excised and fixed in 10% buffered formalin. The fixed samples were embedded in paraffin wax and sectioned perpendicular to the long axis using a microtome to obtain 4.5 µm-thick sections. These thin sections were mounted on glass slides and examined under a fluorescence microscope to visualize the distribution and depth of fluorescence within the skin layers.

However, it is essential to understand that goat ear skin, although widely used for ex vivo permeation studies, does not fully replicate the physiological characteristics of vaginal mucosa. The vaginal epithelium is a non-keratinized stratified squamous tissue without a stratum corneum, covered by a mucus layer, and with a physiological pH of 3.5 to 4.5. In contrast, skin has a keratinized stratum corneum, differs in hydration status and lipid composition, exhibits barrier properties, and has a physiological pH of 5.0. Therefore, the present ex vivo study was interpreted as a comparative assessment of vesicular penetration behaviour rather than a direct predictor of intravaginal absorption kinetics [38,39].

### 2.12. Stability Study

Short-term stability studies were conducted to assess the physicochemical integrity of the optimized liposomal formulation. The formulation was stored in tightly closed glass vials at 4 ± 2 °C for 90 days to minimize the risk of phospholipid hydrolysis and drug leakage associated with room-temperature storage. Samples were collected at predetermined time points (Days 0, 30, 60 and 90) and analyzed for changes in particle size and EE, which are critical indicators of formulation stability. Significant changes in these parameters could indicate degradation, aggregation, or drug loss from the liposomes [40].

### 2.13. Statistical Analysis

All results are presented as mean ± standard deviation (SD) from three independent experiments to ensure reproducibility. Statistical analyses were performed using GraphPad Prism (version 8, GraphPad Software, Inc., La Jolla, CA, USA). The results were analyzed using one-way analysis of variance (ANOVA) to evaluate statistical significance. A *p*-value less than 0.05 (*p* < 0.05) was considered statistically significant, indicating meaningful differences among the experimental groups [41].

## 3. Results and Discussion

### 3.1. Preparation and Characterization of AN-AgNPs

**Figure 1:**
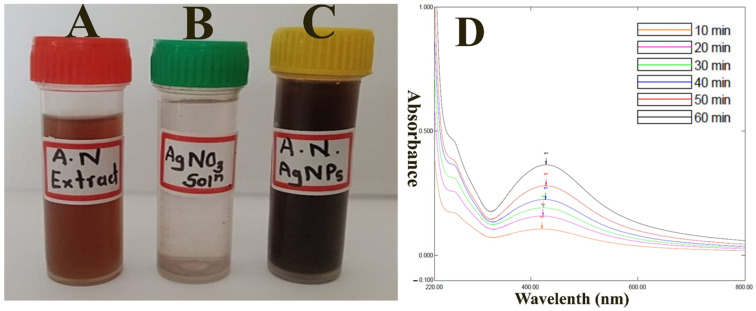
Visual Analysis of (**A**) AN extract, (**B**) AgNO3 and (**C**) AN-AgNPs; (**D**) UV-Visible spectra of ANAgNPs at different time intervals.

The aqueous extract of AN was rich in bioactive phytoconstituents such as phenolics, flavonoids, carbohydrates, proteins, amino acids, and polysaccharides, which played an essential role in the green synthesis, reduction, and stabilisation of AN-AgNPs. The formation of AgNPs was initially evidenced by a change in colour from light brown to dark brown (Figure 1A–C) due to surface plasmon resonance (SPR), and was further confirmed by UV-visible spectroscopy of AN-AgNPs at different time intervals, which showed a characteristic SPR peak at 416 nm, indicative of predominantly spherical nanoparticles (Figure 1D). Although AN extract contains a wide range of phytoconstituents, the reduction of Ag^+^ ions and stabilization of silver nanoparticles (AgNPs) are primarily attributed to a limited number of chemically active components. In particular, phlorotannins (polyphenolic compounds) act as the primary reducing agents due to their multiple hydroxyl groups capable of donating electrons, thereby facilitating the conversion of Ag^+^ to Ag^0^, while sulfated polysaccharides such as fucoidans predominantly function as capping and stabilizing agents through steric and electrostatic interactions.

**Figure 2:**
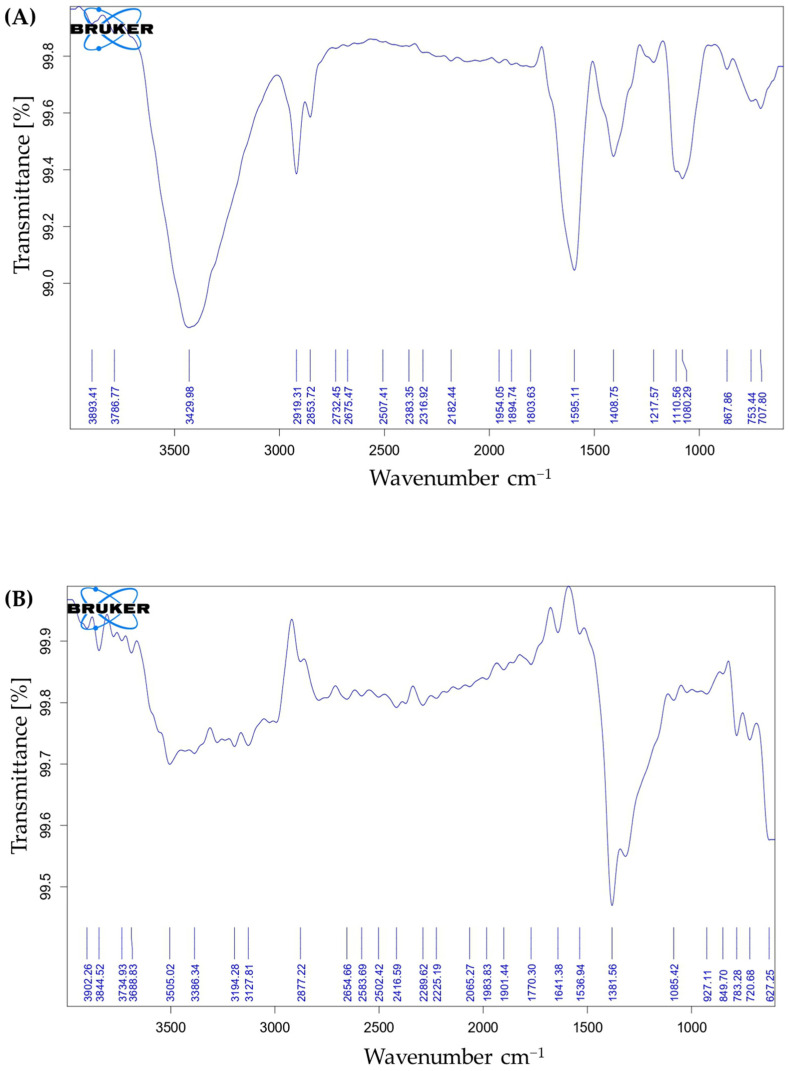

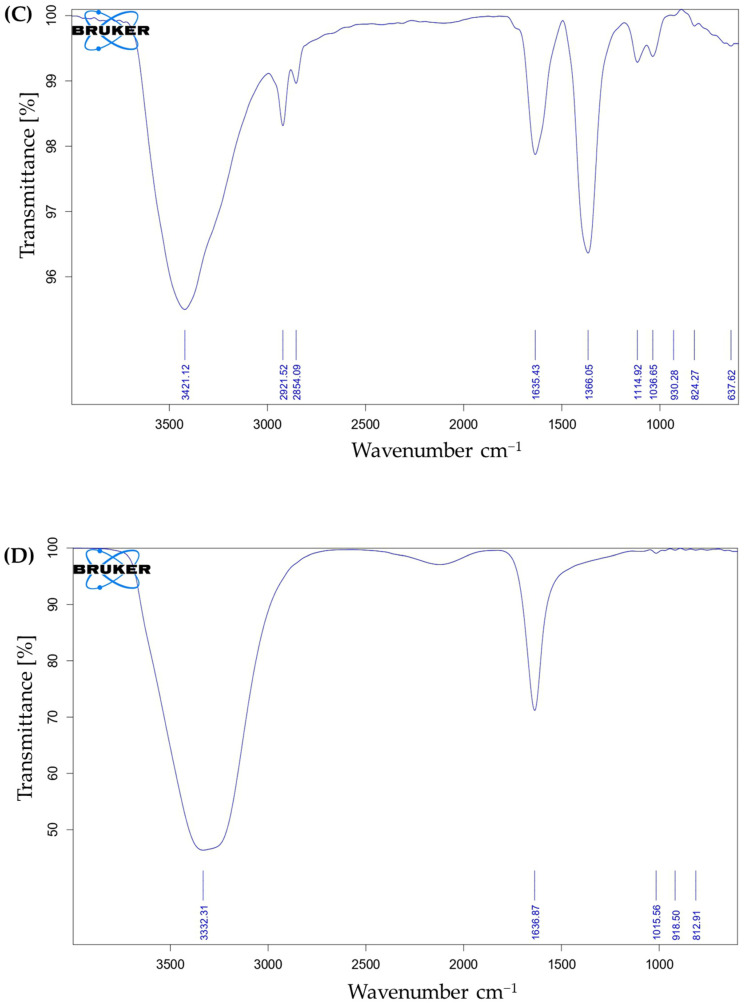
FTIR spectra of (**A**) AN Extract, (**B**) AgNO_3_, (**C**) Physical Mix, (**D**) AN-AgNPs.

This selective involvement was supported by FTIR analysis (Supplementary Table S8), which revealed noticeable shifts in characteristic –OH (3429.98 cm^−1^), C=O (1595.11 cm^−1^), and S=O (1217.57 cm^−1^) functional groups following nanoparticle formation, indicating their direct participation in the redox and stabilization processes. In contrast, other constituents such as vitamins, amino acids, and minerals are less likely to contribute significantly to nanoparticle synthesis and are expected to remain largely in the supernatant [42]. Additionally, FTIR analysis revealed the synthesized silver nanoparticles (AgNPs), raw plant extract, and silver nitrate precursors provides substantial evidence of the reduction and capping process. The AN extract (Figure 2A) exhibited multiple absorption bands corresponding to hydroxyl, aliphatic, carbonyl, and polysaccharide-associated functional groups. A broad band at 3429.98 cm^−1^, along with weaker features at 3786–3893 cm^−1^, corresponds to –OH stretching vibrations of phenolics and alcohols, indicating the presence of polyphenols. Peaks at 2919.31 and 2853.72 cm^−1^ are attributed to aliphatic –CH stretching, while the band at 1595.11 cm^−1^ corresponds to C=O stretching of carbonyl groups from proteins or oxidized phenolics. The region 1217–1080 cm^−1^ corresponds to C–O–C and C–O stretching vibrations in polysaccharides such as fucoidans. Following nanoparticle formation, the FTIR spectrum of AN-AgNPs showed a simplified profile with peaks at 3332.31, 1636.87, and 1015.56 cm^−1^, along with minor bands in the fingerprint region. The shift of the –OH band from 3429.98 to 3332.31 cm^−1^ and the carbonyl band from 1595.11 to 1636.87 cm^−1^, accompanied by reduced intensity, indicates the involvement of these groups in the reduction of Ag^+^ to Ag^0^ via electron donation and subsequent coordination to the nanoparticle surface. The persistence of the 1015 cm^−1^ band confirms the role of polysaccharides in stabilizing AgNPs through steric and electrostatic interactions. Furthermore, the visual transition from a clear solution to a stable precipitate confirms the chemical reduction of silver ions Ag^+^ into metallic nanoparticles Ag^0^, as unreacted silver nitrate remains soluble in the supernatant and does not contribute to the solid-phase FTIR signals. While subsequent HPLC and supernatant analyses could provide a more granular quantification of phytochemical depletion, the current spectroscopic data and physical transformation provide a reliable, peer-validated baseline for confirming successful green synthesis and bioactive capping [43,44].

**Figure 3:**
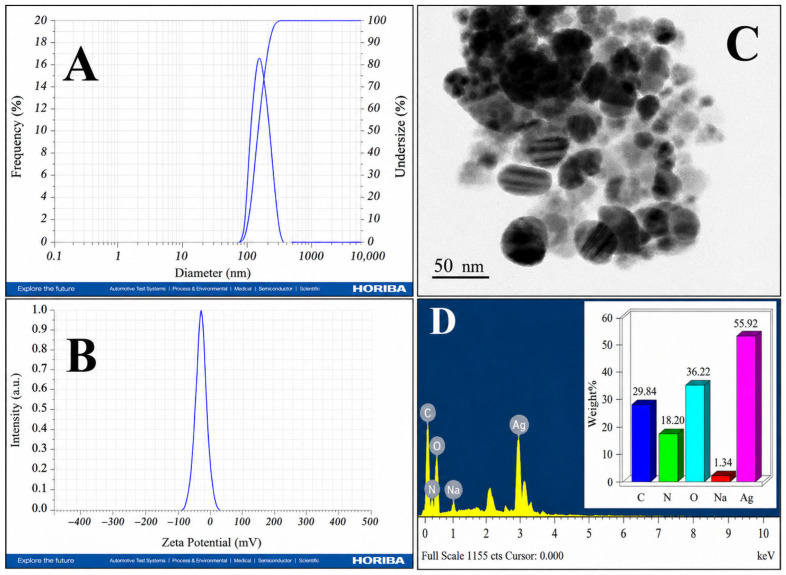
(**A**) Particle size of AN-AgNPs, (**B**) Zeta Potential of AN-AgNPs, (**C**) TEM analysis of AN-AgNPs, (**D**) SEM EDX analysis of AN-AgNPs.

Transmission electron microscopy (TEM) analysis (Figure 3C) confirmed the formation of predominantly spherical AN-AgNPs with a relatively narrow size distribution. Quantitative measurement of over 100 nanoparticles using ImageJ revealed an average particle size of 98.6 ± 18.4 nm, which is smaller than the hydrodynamic diameter obtained from dynamic light scattering (DLS) (143.9 ± 2 nm; PDI 0.420 ± 0.03) (Figure 3A), and a negative zeta potential (−31.5 ± 1.2 mV) (Figure 3B), indicating good colloidal stability imparted by adsorbed phytochemicals. This difference is expected, as DLS measures the hydrodynamic radius including the solvation layer and surface-bound phytoconstituents, whereas TEM reflects the actual metallic core size [45,46]. The TEM micrographs also showed a thin organic capping layer surrounding the nanoparticles, along with some background residues attributable to unbound phytochemicals from the AN extract. These surface-associated biomolecules, primarily polyphenols and polysaccharides, play a crucial role in stabilizing the nanoparticles via steric and electrostatic interactions, which enhances stability and antimicrobial activity, although excess components may remain in the supernatant after synthesis [47]. Elemental analysis by EDX (Figure 3D) confirmed the presence and purity of metallic silver (55.92%) and the coexistence of carbon, nitrogen, and oxygen, indicating effective phytochemical capping and functionalisation and collectively supporting the suitability of AN-AgNPs for biomedical and antifungal applications.

**Figure 4:**
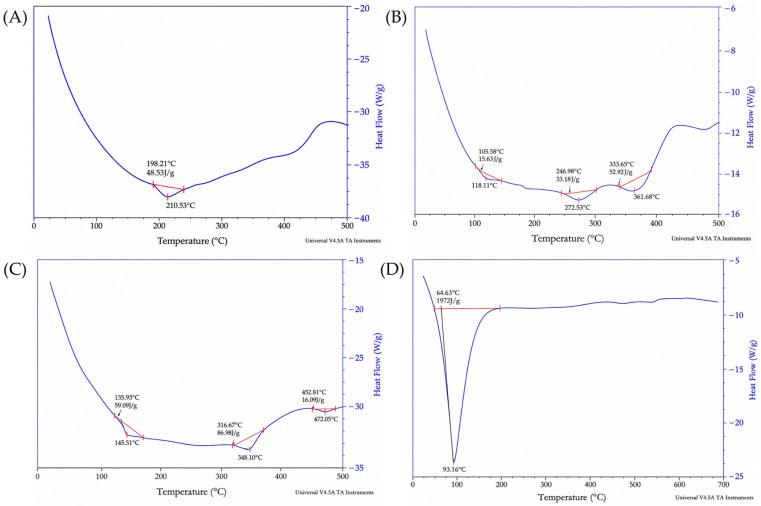
DSC spectra of (**A**) IBC, (**B**) HSPC, (**C**) Cholesterol and (**D**) Liposomes.

In addition, the DSC thermogram of pure IBC (Figure 4A), HSPC (Figure 4B), and Cholesterol (Figure 4C) showed distinct endothermic peaks at 210.53 °C, 118.11 °C, and 145.51 °C, respectively. In contrast, these peaks were not observed in the nanoformulation, suggesting that the drug was encapsulated within the carrier matrix.

### 3.2. Formulation, Optimisation and Characterisation of IBC and AN-AgNP Loaded Liposomes

#### 3.2.1. Drug–Excipient Compatibility Study

**Figure 5:**
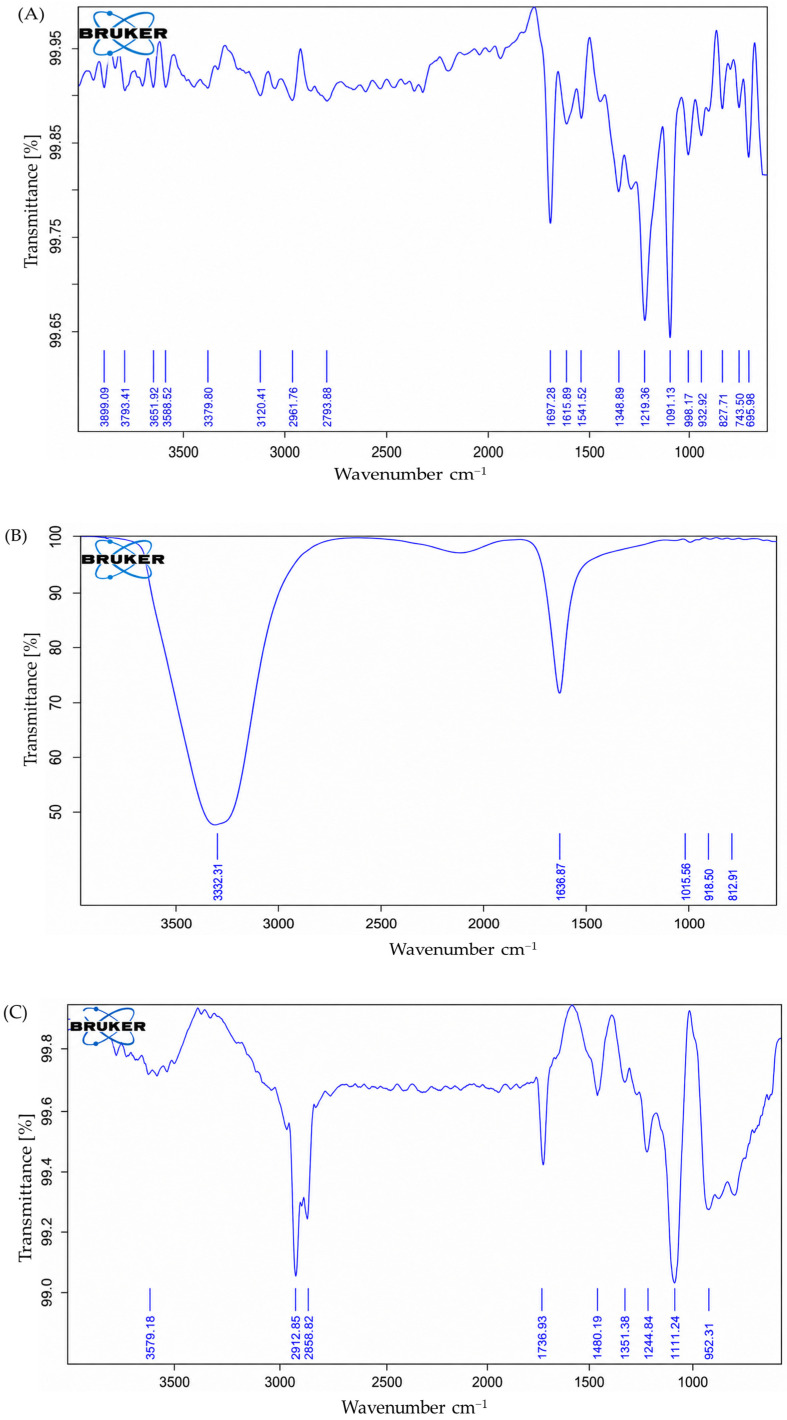

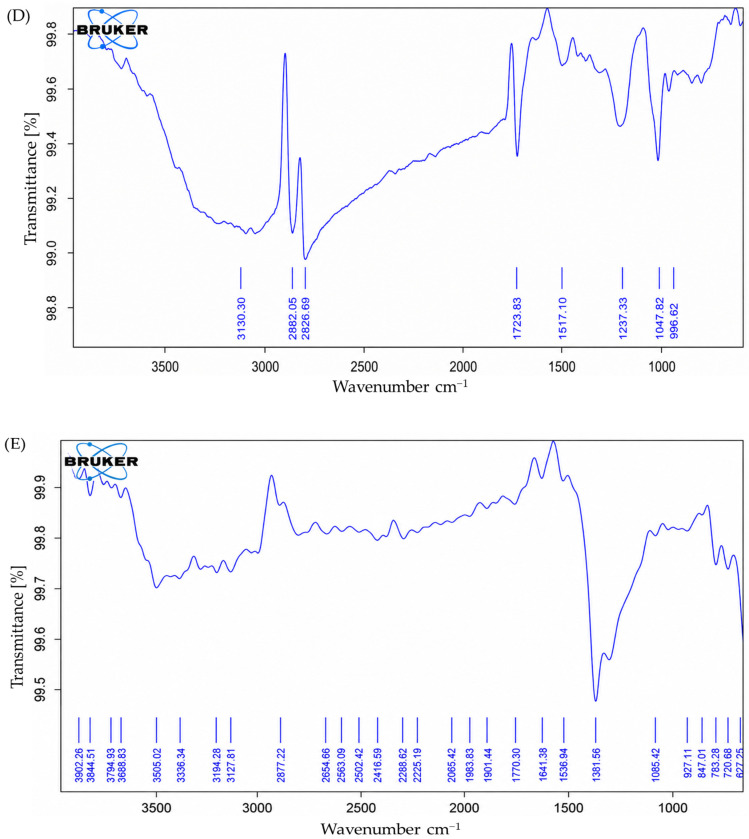
FTIR spectra of (**A**) IBC, (**B**) AN-AgNPs, (**C**) HSPC, (**D**) Cholesterol and (**E**) Liposomes.

FTIR, DSC, and XRD analysis collectively confirmed the successful formation, encapsulation, and stabilization of the IBC-AN-AgNPs co-loaded liposomal formulation. FTIR spectra (Figure 5) showed characteristic peaks of IBC at 3400 cm^−1^ (O-H/N-H), 1700 cm^−1^ (C=O), and 1500–1000 cm^−1^ (C=C, C-N, C-O) (Figure 5A); AN-AgNPs at 3300 cm^−1^ (O-H) and 1600 cm^−1^ (C=C/C=O) (Figure 5B); and typical lipid/sterol bands of HSPC and cholesterol at 2850–2950 cm^−1^ (CH_2_), 1730 cm^−1^ (ester C=O), 1230–1080 cm^−1^ (phosphate), and 1050–1000 cm^−1^ (C-O) (Figure 5C,D).

Merged and shifted peaks in the final formulation confirmed strong molecular interactions and effective encapsulation. DSC thermograms (Figure 4A–C) revealed a sharp melting endotherm of crystalline IBC at 98.74 °C, broad transitions of HSPC at 150–250 °C, and cholesterol at 148–154 °C. Formulated liposomes (Figure 4D) showed a broad, less intense transition at 200–250 °C, with the IBC melting peak disappearing, indicating drug encapsulation, reduced crystallinity, and enhanced thermodynamic stability.

**Figure 6:**
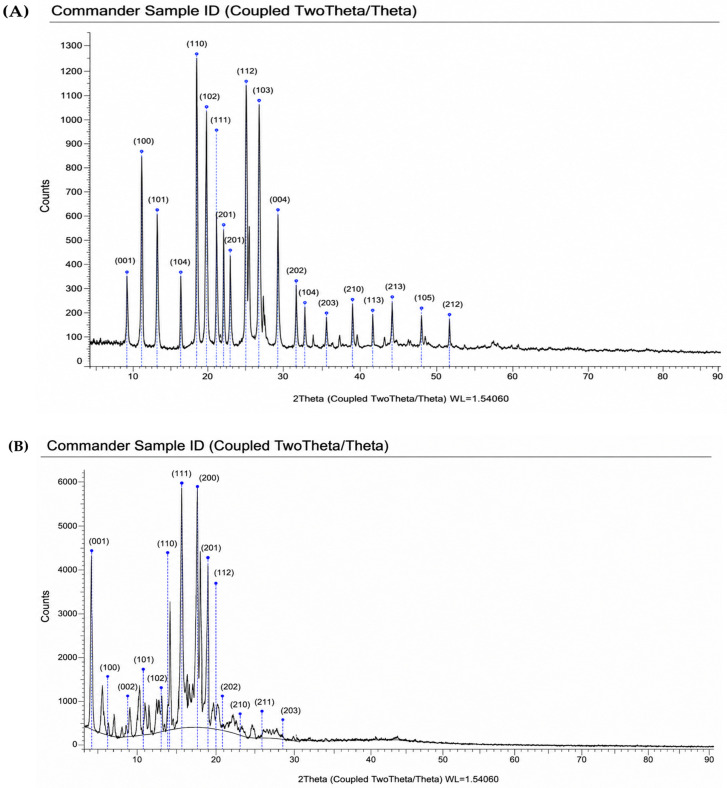

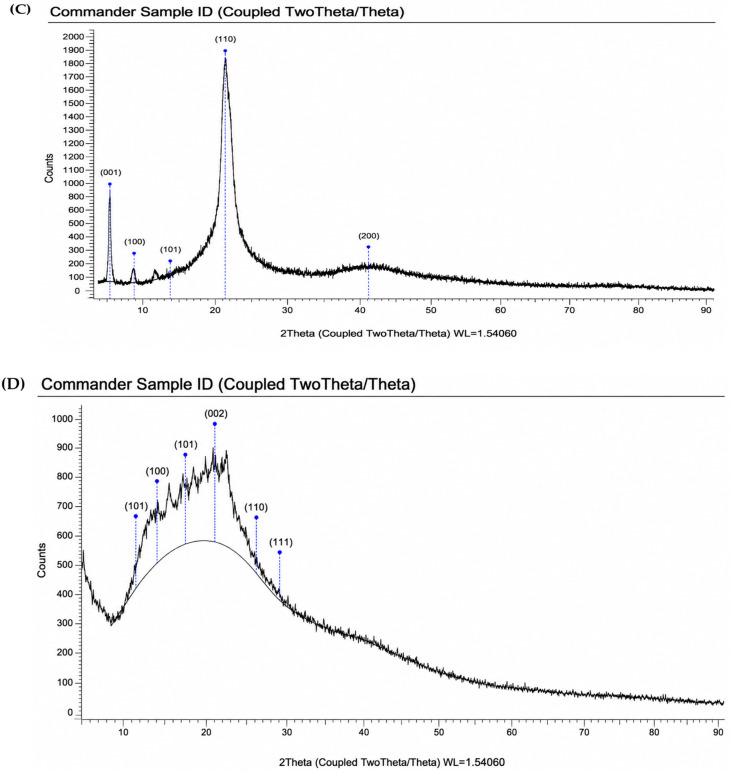
XRD pattern of (**A**) IBC, (**B**) Cholesterol, (**C**) HSPC and (**D**) Liposomes and the Miller indices (hkl) of the peaks.

XRD (Figure 6A–C) further supported these findings, as sharp crystalline peaks of IBC (2θ = 15°, 20°, 22°, 25°), HSPC (8.95°, 12.83°, 21.59°), and cholesterol (14.25 h 18.25°) were replaced by a broad amorphous halo between 10° and 30° (2θ) in the final formulation, confirming conversion to an amorphous state associated with improved solubility and bioavailability [48].

#### 3.2.2. Design of Experiments (DOE)

The IBC liposomes were optimised using Design Expert^®^ software (Version 23) and a two-factor, three-level 3^2^ factorial design. In this optimisation study, particle size and EE were selected as the two key dependent variables. At the same time, the quantity of hydrogenated soy phosphatidylcholine (HSPC) (X_1_) and the amount of cholesterol (X_2_) were chosen as independent variables. Each factor was examined at three levels: low (–1), medium (0), and high (+1). Thirteen experimental runs (Table 1) were conducted to carefully examine the influence of these factors on the chosen responses.

Various mathematical models, including linear, two-factor interaction (2FI), quadratic, and cubic, were applied to the experimental data to ascertain the best fit for forecasting response behaviour. The statistical significance of the models and their terms was assessed using ANOVA. The associations between independent variables and response variables were illustrated using 3D response surface plots and perturbation graphs, all produced by the software [49].

##### Fitting of Data into the Model

Experimental responses from the 13 formulations were analyzed using Design-Expert^®^ software to identify the best appropriate model. Models were evaluated according to higher R^2^, adjusted R^2^, and predicted R^2^ values, as well as lower standard deviation (SD), coefficient of variation (% CV), and predicted residual sum of squares (PRESS) (Supplementary Table S1). A lower PRESS value indicates superior model predictability. According to these criteria, the quadratic model most accurately represented the particle size data, whereas the linear model was optimal for EE.

##### Impact of Independent Variables on the Particle Size (Y_1_) of Liposomes

The reaction can be anticipated for certain levels of each element using the equation expressed in coded factors. The components’ high and low levels are shown by the numerals +1 and −1, respectively, by default. By analyzing the factor coefficients, the coded equation can ascertain the relative significance of the elements.

Table 1 displays the particle sizes of the formulations of IBC and AN-AgNPs-loaded liposomes. The particle size values were between 121 and 159 nm. The subsequent linear equation indicates the influence of the independent variables on the particle.

$$Y_{1}=157.8+0.5494X_{1}-1.63X_{2}+1.28X_{1}X_{2}-15.26X_{12}-6.25X_{22}$$

When X_1_ is the quantity of HSPC, X_2_ is the amount of cholesterol, and Y_1_ is the particle size. According to the equation, particle size is positively affected by HSPC and negatively affected by cholesterol. This shows that as the quantity of HSPC supplied increases, the particle size increases, whereas as the amount of cholesterol added increases, the particle size decreases. The high X_2_ coefficient indicates that the amount of HSPC has a greater impact on the liposomes’ particle size.

Supplementary Table S2 presents the ANOVA outcomes for the particle size data. The model’s importance is demonstrated by its F-value of 4.38. The probability of a Model F-Value of this significance arising from random noise is about 3.98%. Model terms are deemed significant if their “Prob > F” values are below 0.0500. X_1_ and X_2_ are significant variables in this context. The Adjusted R-Squared of 0.5846 and the Predicted R-Squared (R2) of 0.6720 exhibit a reasonable concordance. The signal-to-noise ratio is assessed with sufficient accuracy. A ratio higher than four is preferred. Your signal strength of 5.111 is sufficient. This paradigm can be used to navigate the design space.

**Figure 7:**
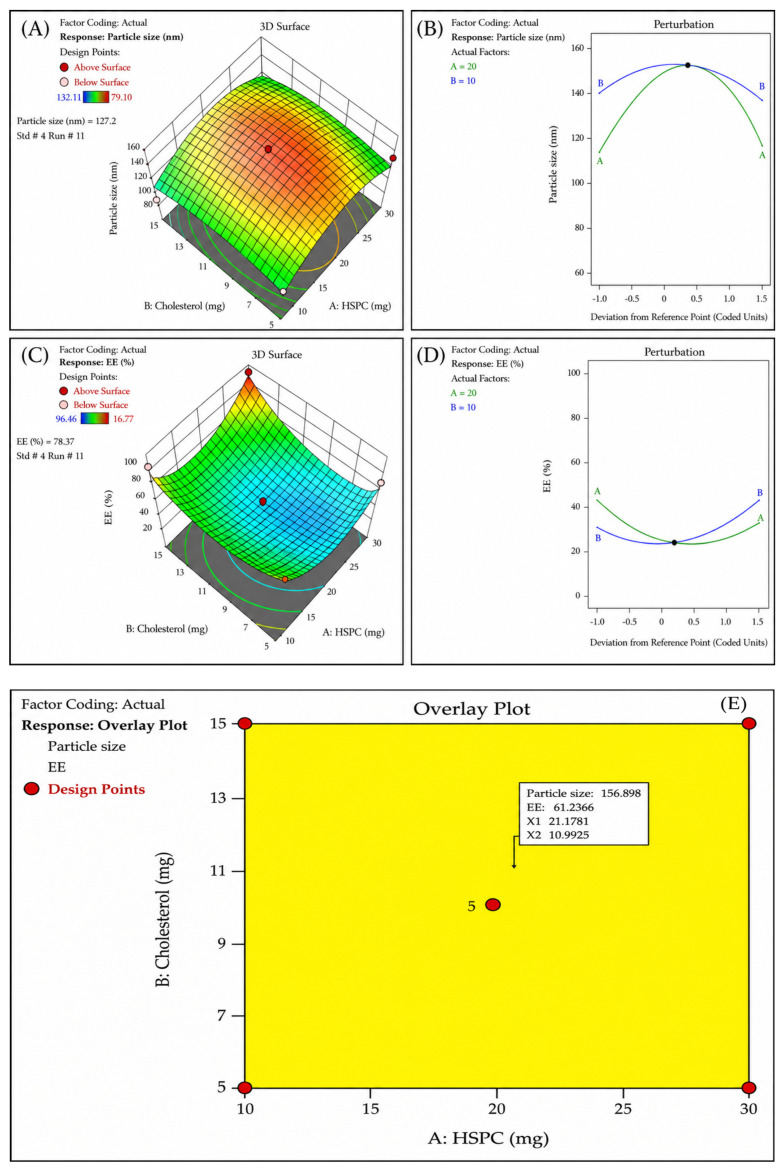
(**A**) 3D-response surface curve of variable particle size (X1), (**B**) Perturbation plot of variable particle size (X1), (**C**) 3D-response surface curve of variable EE (X2), (**D**) Perturbation plot of variable EE (X2), and (**E**) Displaying the anticipated response values and the optimum Liposome formulation in the design space. 3D response-surface and Perturbation graphs showing the effect of HSPC and Cholesterol on Particle size and Entrapment Efficiency.

3D response-surface graphs (Figure 7A) corresponding to them show how the independent factors affect particle size. The charts and plots show that as the HSPC concentration increased from 10 mg to 30 mg, the particle size also increased. These findings were corroborated by the particle perturbation plot (Figure 7B). The amount of cholesterol was a key factor influencing particle size, as indicated by the perturbation plot’s steep slope for factor B (amount of cholesterol) and a modest bend for factor A (amount of HSPC).

##### Impact of Independent Variables on the EE (Y_2_) of Liposomes

Quantitative values for each element, along with forecasts for the reaction, can be derived from the equation stated in terms of coded variables. The components’ high and low levels are shown by +1 and −1, respectively, by default. By analyzing the factor coefficients, the coded equation can ascertain the relative significance of the elements. Table 1 displays the EE of the liposome formulations. The EE values ranged from 55% to 76%. The subsequent quadratic equation elucidates the influence of the independent variables on the EE.

$$Y_{2}=60.42-1.49X_{1}+1.49X_{1\;}+3.19X_{2\;}+3.84X_{1}X_{2}+6.08X_{12}+4.43X_{2}$$

where X_1_ is the amount of HSPC, X_2_ is the amount of cholesterol, and Y_2_ is the EE. According to the equation, EE is positively affected by HSPC and negatively affected by cholesterol. This indicates that as HSPC increases and cholesterol decreases, EE also increases. As indicated by the high coefficient value of X_1_, the amount of cholesterol has a greater impact on the entrapment effectiveness of liposomes than does the amount of HSPC. Supplementary Table S2 displays the ANOVA findings for the EE data.

The significance of the model is indicated by its F-value of 4.69. The likelihood that an F-value this significant might be caused by noise is merely 3.38%. The presence of substantial model terms is indicated by a *p*-value (Prob > F) below 0.0500. The X_1_ and X_2_ parameters are essential in this instance. There is reasonable agreement between the Adjusted R2 of 0.6056 and the Predicted R2 of 0.5549. The signal-to-noise ratio is measured with adequate precision. A ratio higher than four is preferred. A sufficient signal is indicated by your ratio of 5.068. This model can be used to traverse the design space.

Figure 7 displays the 3D response surface graphs (Figure 7C) that illustrate how the independent variables affect EE. The charts and plots show that when the amounts of HSPC and Cholesterol were increased from 10 to 30 mg and from 5 to 15 mg, respectively, EE increased. Another tool for understanding how separate factors affect responses is a perturbation plot (Figure 7D).

#### 3.2.3. Optimization of IBC-AN-AgNPs Loaded Liposome Formulation

A numerical optimization approach was used to identify the optimized IBC-loaded liposome formulation within the design space by setting constraints to minimize particle size and maximize EE. The software suggested an optimized formulation (IBCL-11) with 15 mg of HSPC and 24.07 mg of cholesterol, yielding a desirability value of 1. The overlay plot depicts the optimized formulation and its anticipated response values (Figure 7E). The optimized IBC-liposome formulation (IBCL-11) was developed and experimentally assessed for the chosen responses. Supplementary Table S3 illustrates the validation of the optimized formulation by contrasting the expected values derived from the optimization model with the empirically observed responses. The similarity between predicted and observed values validates the reliability and predictive efficacy of the optimization method. The projected particle size was 136.27 nm, whereas the experimentally measured particle size was 127.2 nm, resulting in a percentage error of −6.65%. The anticipated entrapment efficiency was 76.47%, whereas the actual measurement was 76.77%, yielding a minimal percentage error of +0.39%. The minimal percentage errors for both responses affirm the reliability of the optimisation process and verify that the established model is appropriate for predicting essential quality features of the IBC-loaded liposomes formulation.

#### 3.2.4. Characterization of Liposomes

##### Particle Size and Zeta Potential

**Figure 8:**
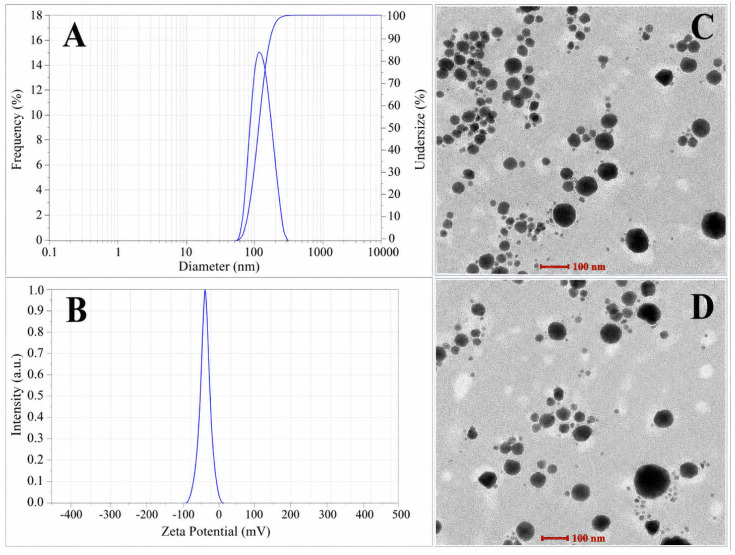
(**A**) Particle size of IBCL-11 liposomal formulation, (**B**) Zeta Potential of IBCL-11 liposomal formulation, (**C**,**D**) TEM analysis of IBCL-11 liposomal formulation.

The optimized IBCL-11 liposomal formulation exhibited the smallest vesicle size among all evaluated formulations, with an average diameter of 127.2 ± 3.4 nm (Figure 8A) Nanoparticles in this size range are typically regarded as more beneficial for drug delivery, as they can facilitate enhanced permeability and retention (EPR) effects, boost cellular internalization, and improve biodistribution. Smaller vesicles also augment the surface area-to-volume ratio, hence improving drug loading and interaction with biological membranes. A zeta potential study was conducted for IBCL-11 to evaluate its colloidal stability. The measured zeta potential of −43.8 ± 2.5 mV (Figure 8B) indicates that the formulation has enough negative surface charge to inhibit particle aggregation via electrostatic repulsion. A zeta potential magnitude of ±30 mV is generally considered indicative of favourable physical stability, crucial for preserving the homogeneity and shelf life of nanoparticulate systems. The precise characterisation of IBCL-11 with respect to particle size and surface charge underscores its advantageous physicochemical properties, indicating its potential for effective and stable therapeutic administration [50].

##### Entrapment Efficiency

The EE of the optimised liposomal formulation (IBCL-11) was quantified using the indirect centrifugation technique. The experimentally observed EE was 76.77%, closely matching the theoretical value of 76.47% predicted by the DOE software (Version 23) (Supplementary Table S4). The close agreement between the predicted and observed values (percentage error < 0.5%) substantiates the validity and robustness of the mathematical model used to optimise the formulation parameters. The high % EE (approximately 77%) indicates successful intercalation of Ibrexafungerp Citrate (IBC) within the lipid bilayer of the liposomes. Furthermore, the indirect measurement method, confirmed by UV-spectrophotometric analysis of the supernatant at 286 nm, provided a reliable and reproducible means of estimating the unentrapped drug fraction [51].

##### Surface Morphology of TEM

TEM analysis was performed to ensure the formation of IBC-loaded liposomes and to examine their surface morphology and structural attributes. The TEM images (Figure 8C,D) displayed spherical to nearly spherical unilamellar vesicles exhibiting a modest size distribution. The liposomes displayed distinct boundaries, signifying the successful production of complete bilayer structures. No notable aggregation or deformation was seen, indicating robust colloidal stability of the formulation. The lack of aggregation signifies the colloidal stability of the IBC-liposomes [52].

### 3.3. Formulation and Characterization of Liposomal Gel

The liposomal gel was successfully formulated using Carbopol-934 and CMC as gelling agents, yielding a smooth, homogeneous dispersion following neutralisation with TEA. Incorporation of the optimised liposomal formulation into the gel base was achieved without phase separation, indicating good compatibility between the liposomes and the polymeric matrix. The addition of propylene glycol further enhanced the formulation’s uniformity and is expected to facilitate drug permeation across the vaginal mucosa. The optimized gel formulation appeared as a homogeneous brown dispersion with a drug content of 90%. The pH was 6.3, consistent with the physiological pH of skin, indicating minimal risk of irritation. Viscosity analysis using a Brookfield viscometer yielded a value of 6842 cP, demonstrating rheological properties suitable for topical application [53].

### 3.4. In Vitro Drug Release Profile

**Figure 9:**
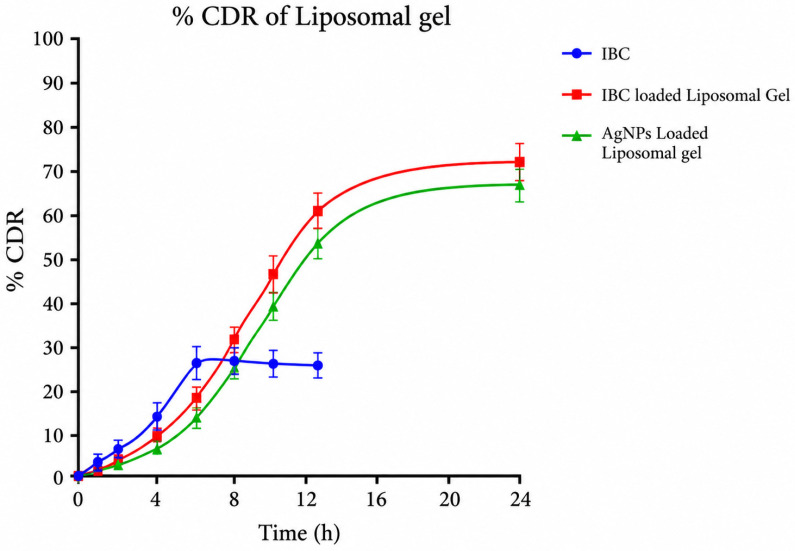
In vitro drug release study of Pure IBC, IBC-loaded Liposomal gel, and AgNP-loaded liposomal gel.

The in vitro drug release profile was evaluated over 24 h to assess the formulation’s sustained-release potential (Figure 9). Pure IBC (API) exhibited a rapid initial release, reaching approximately 24.54% within 12 h and plateauing thereafter, indicating limited sustained-release capability (Supplementary Table S4). In contrast, the IBC-loaded liposomal gel exhibited a gradual, extended release profile, with only 1.7% released at 0.5 h and 72.6% released by 24 h, demonstrating significant sustained release behavior. The AN-AgNP-loaded liposomal gel showed the slowest initial release (0.02% at 0.5 h), followed by a gradual increase, reaching 69.18% at 24 h. This prolonged release from both liposomal formulations is attributed to the encapsulation of the drug and nanoparticles within the phospholipid bilayers and gel matrix, which act as controlled-release reservoirs. Notably, the AN-AgNP liposomal gel exhibited slightly slower release kinetics in the early phases compared to the IBC-only liposomal gel, likely due to additional matrix complexity and nanoparticle interactions, suggesting enhanced control over drug diffusion. These findings confirm the effectiveness of liposomal systems in sustaining and modulating IBC release, offering potential therapeutic advantages for the topical treatment of VVC [54,55].

### 3.5. In Vitro Antifungal Study

#### 3.5.1. MIC and MFC of AN, IBC, AN-AgNPs and Liposomes

The conventional broth dilution method was used to assess the antifungal activity of AN, the IBC drug, AN-AgNPs, and optimized IBCL-11 liposomes against *C. albicans* and *C. glabrata*. The IBC drug served as the reference. Significant inhibitory effects were observed for AN, IBC, AN-AgNPs, and optimized IBCL-11. Table 2 presents the MIC results for each test sample. For *C. albicans*, the MIC values were 125 µg/mL for AN, eight µg/mL for IBC, 62.5 µg/mL for AN-AgNPs, and less than one µg/mL for liposomes (Supplementary Figure S1). For *C. glabrata*, the MIC values were 31.25 µg/mL for AN-AgNPs, eight µg/mL for liposomes, 250 µg/mL for AN, and eight µg/mL for IBC (Supplementary Figure S2). Notably, optimized IBCL-11 liposomes had the lowest MIC (<1 µg/mL) against *C. albicans*, indicating greater antifungal potency than AN, AN-AgNPs, and pure IBC.

**Figure 10:**
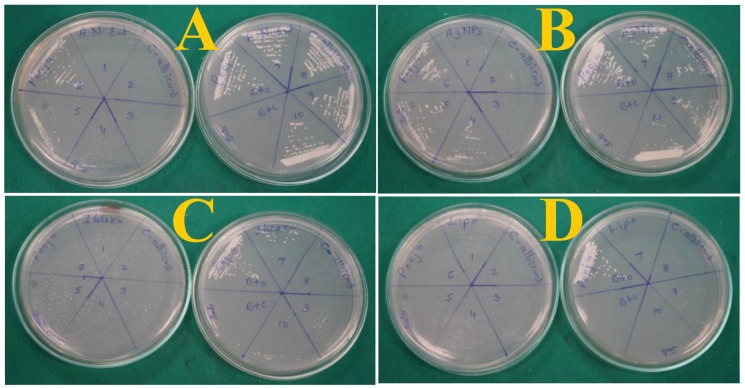
MFC of (**A**) AN extract (**B**) IBC drug (reference) (**C**) AN-AgNPs (**D**) Liposomes on *C. albicans.* (In each study, the first Petri dish was divided into 1 to 6 zones and second Petri dish represents 7 to 10 zones).

**Figure 11:**
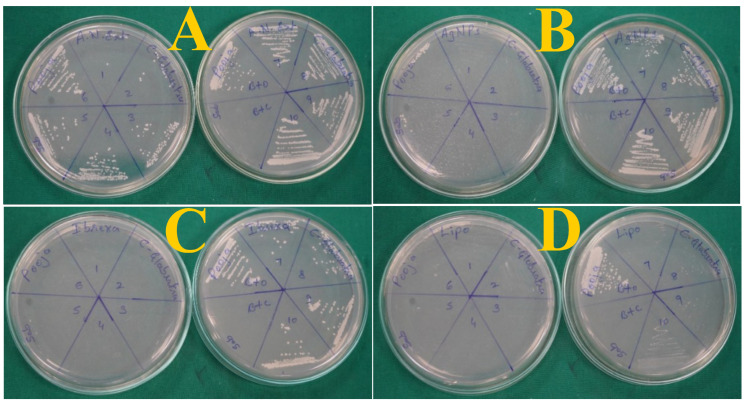
MFC of (**A**) AN extract (**B**) IBC drug (reference) (**C**) AN-AgNPs (**D**) Liposomes on *C. glabrata*. (In each study, the first Petri dish was divided into 1 to 6 zones and second Petri dish represents 7 to 10 zones).

The concentration required to rapidly eradicate 99.9% of the original inoculum is known as the MFC. As reported in Table 2, AN, IBC, AN-AgNPs, and optimized IBCL-11 Liposomes had MFC values of 250 µg/mL, 16.12 µg/mL, 125 µg/mL, and less than 1 µg/mL, respectively, against *C. albicans* (Figure 10). The MFC values (Table 2) for AN, IBC, AN-AgNPs, and optimized IBCL-11 Liposomes were >500 µg/mL, 16.12 µg/mL, and 62.5 µg/mL, respectively, for *C. glabrata* (Figure 11). Interestingly, Liposomes showed the lowest MFC value (<1 µg/mL) against *C. albicans*, suggesting they are more effective at killing the fungus than AN.

The present investigation evaluated the fungicidal or fungistatic properties of AN, the IBC drug, AN-AgNPs, and optimized IBCL-11 Liposomes against *C. albicans* and *C. glabrata* at different doses. The IBC drug was used as a reference. Liposome treatment completely inhibited *C. albicans* growth, with an effect more effective than that on *C. glabrata*. Furthermore, the Liposome treatment had a considerably lower number of colony-forming units than the AN, IBC, and AN-AgNP treatments. Given these effects on VVC, liposomes have demonstrated superior antifungal efficacy against *C. albicans* compared with *C. glabrata* 58.

#### 3.5.2. Time–Kill Kinetic Assay

**Figure 12:**
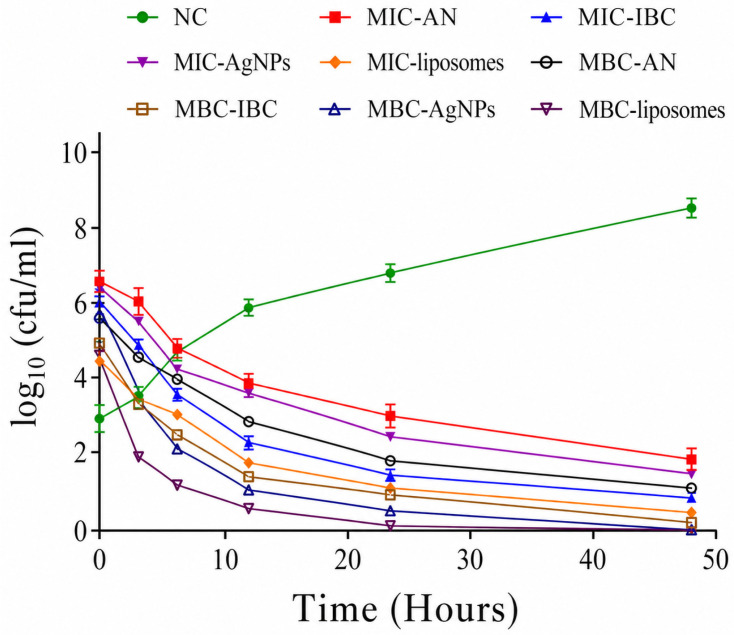
Time kill curve showing time-dependent bacterial inhibition and fungicidal activity of AN-extract, free IBC, and AN-AgNPs and Liposomes on *C. albicans*. Data are mean ± SD of three independent experiments. MIC: minimum inhibitory concentration; MFC: minimum fungicidal concentration.

The time kill kinetics of AN-extract, IBC, AN-AgNPs, and their liposomal formulation (IBC + AN-AgNP-loaded liposomes) were evaluated against *Candida albicans*, selected based on its highest susceptibility (lowest MIC and MFC values). This approach enabled a robust assessment of fungicidal dynamics and time-dependent killing behaviour of the developed nano formulation. As illustrated in Figure 12, the liposomal formulation exhibited rapid and sustained fungicidal activity, achieving complete eradication of *C. albicans* at the MFC within 48 h. In contrast, AN-extract, free IBC, and AN-AgNPs demonstrated comparatively slower killing kinetics with incomplete fungal clearance over the same duration. At MIC levels, all treatments showed time-dependent inhibition; however, the liposomal system consistently produced a greater log reduction in viable counts across all time points. The superior antifungal efficacy of the liposomal formulation can be attributed to multiple synergistic mechanisms. Liposomes enhance drug solubilization and facilitate improved interaction with fungal cell membranes, promoting efficient intracellular delivery of IBC and sustained release of bioactive phytoconstituents and AgNPs [56]. IBC, a glucan synthase inhibitor, disrupts fungal cell wall biosynthesis, while AgNPs induce membrane damage, reactive oxygen species (ROS) generation, and intracellular protein dysfunction, resulting in irreversible cellular damage 61. The integration of these components within a lipid bilayer system likely enhances membrane fusion and retention at the fungal surface, thereby amplifying fungicidal kinetics. Additionally, the observed time-dependent killing suggests a concentration- and exposure-driven pharmacodynamic profile, consistent with antifungal agents targeting cell wall integrity and membrane function [57]. The rapid decline in viable fungal counts with liposomes indicates a transition from fungistatic to fungicidal activity, which is critical for preventing recurrence and resistance development. A limitation of the present study is the exclusive evaluation against *C. albicans*. Inclusion of non-*albicans* Candida species such as *C. glabrata* in future studies would provide broader translational relevance, particularly considering their intrinsic resistance patterns.

#### 3.5.3. Biofilm Reduction—Crystal Violet Assay

**Figure 13:**
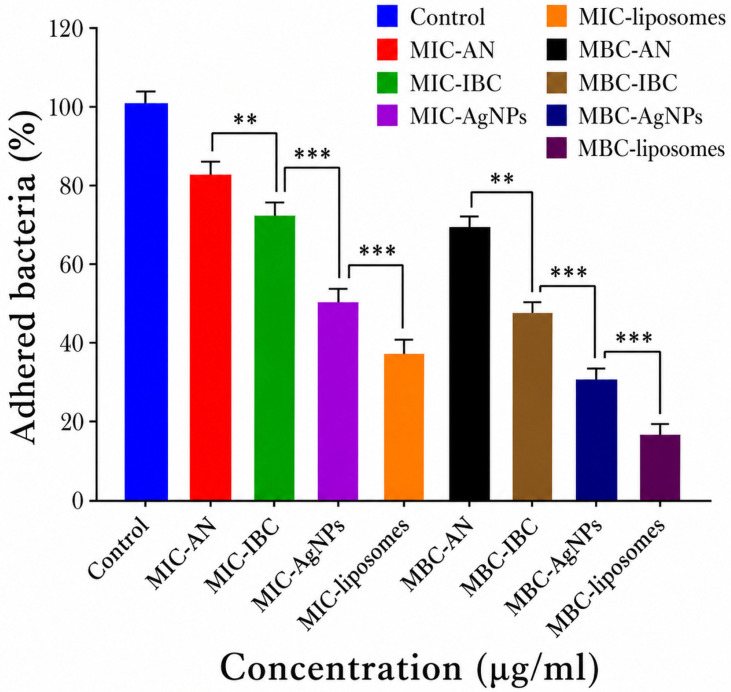
Effect of AN-extract, free IBC, and AN-AgNPs and Liposomes on the fungal inhibition on the microtitre plate. Data are mean ± SD of three independent experiments. Significant difference specified as *** *p* < 0.001, ** *p* < 0.01, between control versus treated samples.

Biofilm-associated *C. albicans* infections are inherently recalcitrant to antifungal therapy due to the presence of a dense extracellular polymeric matrix (EPM), which restricts drug penetration, facilitates quorum sensing, and promotes phenotypic resistance [58]. The antibiofilm efficacy of AN-extract, IBC, AN-AgNPs, and their liposomal formulation was quantitatively evaluated using the crystal violet assay at MIC and MFC levels. As depicted in Figure 13, all treatments exhibited concentration-dependent antibiofilm activity; however, the liposomal formulation demonstrated significantly superior biofilm eradication compared to AN-extract, free IBC, and AN-AgNPs (*p* < 0.001). Importantly, the reported percentages correspond to residual biofilm biomass, indicating that lower values reflect higher antibiofilm efficacy. Quantitative analysis revealed that AN-extract treatment resulted in residual biofilm biomass of 81.24 ± 4.57% (MIC) and 67.31 ± 3.85% (MFC), corresponding to modest biofilm reduction. IBC exhibited improved activity with residual biomass of 70.06 ± 3.42% (MIC) and 46.52 ± 2.53% (MFC), consistent with its mechanism of inhibiting β-(1,3)-D-glucan synthesis in fungal cell walls. AN-AgNPs showed moderate antibiofilm effects, with residual biomass values of 45.27 ± 3.16% (MIC) and 27.62 ± 2.13% (MFC), likely due to nanoparticle-induced oxidative stress and disruption of cell membrane integrity. Notably, the liposomal formulation achieved the lowest residual biofilm biomass, with values of 34.25 ± 2.43% (MIC) and 16.28 ± 1.72% (MFC), indicating ~65–84% biofilm reduction, thereby confirming its markedly enhanced antibiofilm potential. In contrast, the untreated control showed no significant reduction in biofilm mass. The superior antibiofilm activity of the liposomal system can be attributed to its multifunctional and synergistic mode of action. Liposomes facilitate enhanced penetration into the biofilm matrix due to their nanoscale size and lipid bilayer compatibility, enabling efficient delivery of both IBC and AN-AgNPs to deeply embedded fungal cells [59]. IBC disrupts biofilm structural integrity by inhibiting glucan synthesis, while AgNPs induce reactive oxygen species (ROS) generation, protein denaturation, and membrane destabilization, collectively leading to biofilm disintegration [60]. Additionally, phytoconstituents from AN may interfere with quorum sensing and adhesion processes, further weakening biofilm formation. This combinatorial strategy effectively targets multiple stages of biofilm development, including adhesion, maturation, and maintenance, thereby overcoming intrinsic resistance mechanisms associated with sessile fungal communities. Such multi-targeted antibiofilm activity is particularly critical for managing chronic and recurrent Candida infections, where conventional monotherapy often fails. Overall, the findings highlight that liposomal co-delivery of IBC and AN-AgNPs significantly enhances antibiofilm efficacy, positioning this formulation as a promising therapeutic approach for biofilm-associated fungal infections.

### 3.6. Ex Vivo Skin Permeation Profile

**Figure 14:**
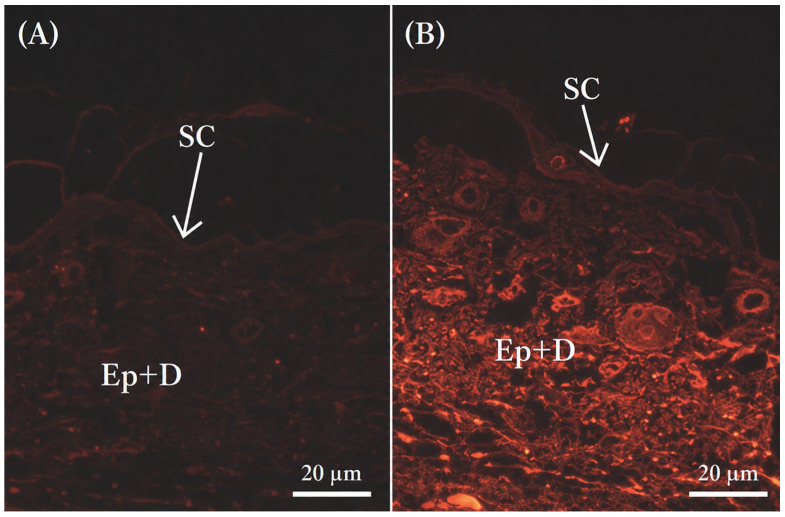
Goat skin fluorescence photos taken 8 h after applying (**A**) Rh6G solution and (**B**) Rh6G-loaded Liposomes. The diffusion of fluorescence into SC and Ep + D skin layers is seen in images. (SC—Stratum Corneum layer; Ep + D—Epidermal and Dermal layers).

Fluorescence microscopy was used to compare the interaction of free Rh6G and Rh6G-loaded liposomes with the biological barrier after ex vivo application to goat ear skin mounted in a Franz diffusion cell. Following eight hours of treatment with Rh6G solution (control) and Rh6G-Liposomes, fluorescence microscopy images of goat ear skin are shown in Figure 14. Application of the free Rh6G solution produced fluorescence predominantly in the superficial layers of the tissue, largely confined to the stratum corneum (Figure 14A). Fluorescence microscopy (Figure 14B) revealed a widespread distribution of Rh6G throughout the epidermal and dermal layers after treatment with Rh6G-Liposomes. The detected fluorescence appeared diffuse rather than particulate, owing to the limited resolution of the standard fluorescence microscope, which cannot visualize individual vesicles approximately 120 nm in size. Consequently, distinct liposomal structures cannot be identified in tissue sections. Unlike the control, which showed fluorescence confined to the superficial stratum corneum, the optimized liposomal formulation enabled deep tissue penetration. This suggests that penetration likely involves the adsorption and binding of Rh6G-Liposomes to stratum corneum lipids [61]. Liposomes often do not remain intact as perfect spheres when they enter the skin. Instead, they fuse with skin lipids, rupture, and release their payload (the drug and the dye) into deeper skin layers. This ‘depot effect’ is highly advantageous for treating deep-seated fungal infections, as it ensures a high local concentration of the antifungal agent (IBC and AN-AgNPs) within the target tissue, regardless of whether the carrier remains fully intact. Moreover, the enhanced tissue distribution observed with the liposomal system suggests improved local deposition [62]. Consequently, this may translate into prolonged antifungal efficacy within the vaginal microenvironment, thereby effectively eradicating Candida species while minimizing systemic exposure [63].

### 3.7. Stability Assessment

**Figure 15:**
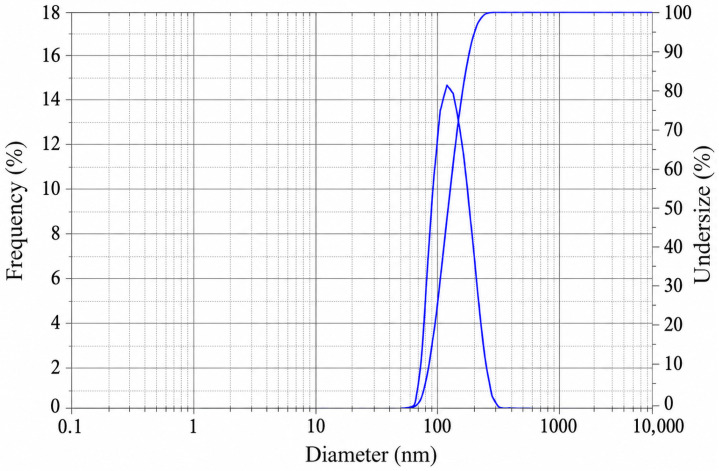
Particle size of Liposomes after 90 days of storage. The solid blue curve represents the differential frequency (%) of the particle population, and the corresponding cumulative undersize (%) is shown on the secondary y-axis. The cumulative undersize curve reached 100% well below 1000 nm, and there was a complete absence of secondary peaks in the larger size ranges. This lack of larger particulate populations indicates that no significant vesicle fusion or aggregation occurred over the 90 days.

The stability of the optimised liposomal formulation was evaluated over 90 days at refrigerated conditions (4–8 °C) by monitoring EE and particle size, as shown in Table 3. Initially, the formulation showed an EE of 76.77 ± 0.53% and a particle size of 127.2 ± 1.23 nm. After 30 days, slight changes were observed in EE (76.12 ± 0.37%) and particle size (127.89 ± 1.36 nm). By day 60, the values were 75.70 ± 0.46% and 128.3 ± 1.05 nm, respectively. After 90 days, the values were 75.70 ± 0.46% and 128.8 ± 1.05 nm for EE and particle size, respectively (Figure 15). These minimal variations indicate good physical stability of the formulation, with negligible drug leakage or aggregation over time, making it suitable for extended storage.

## 4. Conclusions

The present study developed and assessed a novel, eco-friendly liposomal formulation co-loaded with green-synthesized AN-AgNPs and IBC for the targeted topical therapy of VVC. The biosynthesized AN-AgNPs demonstrated favorable nanoscale dimensions, steady zeta potential, and significant antifungal efficacy. The liposomal formulation exhibited enhanced drug EE, consistent nanometric particle size, and robust physical stability under refrigeration for 90 days. In vitro antifungal experiments demonstrated markedly improved fungicidal efficacy against *C. albicans* and *C. glabrata* in comparison to the free drug and nanoparticles individually. Skin permeation and in vitro release experiments demonstrated enhanced mucosal penetration and sustained drug release, facilitating prolonged local action at the infection site. This co-loaded liposomal gel signifies a promising, tailored nanomedicine strategy for the effective treatment of resistant and recurrent VVC. Additional in vivo studies and systematically organized clinical trials are necessary to confirm its safety, therapeutic efficacy, and patient acceptability for the potential clinical use.

## 5. Patents

We filed a patent application for this work, which was published on 1 August 2025 (Application No. 202521069554 A).

## Figures and Tables

**Table 1:** The 2-factor Central Composite Design (CCD) matrix showing the 13 experimental runs, coded factor levels, and observed responses.

Batch No	X_1_ HSPC (mg)	X_2_ Cholesterol (mg)	Y_1_ Particle Size (nm)	Y_2_ % EE
L1	0	0	159.32 ± 4.35	59.84 ± 2.54
L2	0	0	158.34 ± 4.95	60.29 ± 3.24
L3	0	0	160.25 ± 5.32	58.32 ± 2.16
L4	+1	0	128.64 ± 3.24	74.45 ± 3.49
L5	−1	0	130.23 ± 3.84	73.66 ± 3.57
L6	−1	+1	121.3 ± 3.18	75.62 ± 3.86
L7	0	0	154.94 ± 4.52	62.52 ± 2.94
L8	0	+1	156.23 ± 5.16	70.26 ± 4.65
L9	0	−1	138.63 ± 4.27	71.25 ± 4.16
L10	+1	+1	143.6 ± 4.68	55.64 ± 2.85
L11	+1	+1	127.26 ± 3.57	76.77 ± 4.26
L12	0	0	155.32 ± 4.64	61.12 ± 3.25
L13	−1	−1	148.83 ± 5.57	69.84 ± 3.76
Factors		Coded level and actual value		
Independent variables		Low (−1)	Medium (0)	High (+1)
Factor X_1_: Amount of HSPC (mg)		10	20	30
Factor X_2_: Amount of Cholesterol (mg)		5	10	15
Dependent Variables or Responses		Constraint		
Response Y_1_: Particle size (nm)		Minimize		
Response Y_2_: Entrapment Efficiency (%)		Maximize		

**Note:** The design matrix consists of 13 experimental runs generated by the Central Composite Design (CCD). X_1_: Factor 1, HSPC (mg); X_2_: Factor 2, Cholesterol (mg). Y_1_: Response 1, Particle Size (nm); Y_2_: Response 2, % EE. Coded levels represent: −1 (Low), 0 (Medium), and +1 (High).

**Table 2:** MIC and MFC value of AN extract, IBC drug (reference), AN-AgNPs and Liposomes on *C. albicans* and *C. glabrata*.

Sr. No.	Test Material	MIC (µg/mL)	MFC (µg/mL)
	*C. albicans*		
1	AN extract	125 ± 5.2	250 ± 6
2	IBC	8 ± 1.5	16.12 ± 2.8
3	AN-AgNPs	62.5 ± 4.7	125 ± 4
4	Liposomes	<1 ± 0.8	<1 ± 0.8
	*C. glabrata*		
1	AN extract	250 ± 6	>500 ± 8
2	IBC	8 ± 1.5	16.12 ± 2.8
3	AN-AgNPs	31.25 ± 3	62.5 ± 4.7
4	Liposomes	8 ± 1.5	8 ± 1.5

**Table 3:** Effect on entrapment efficiency and particle size for liposomal dispersion from liposomal formulation during stability.

Sr. No	No. of Days	Entrapment Efficiency (%)	Particle Size (nm)
		4–8 °C	4–8 °C
1	0	76.77 ± 0.53	127.2 ± 1.23
2	30	76.12 ± 0.37	127.89 ± 1.36
3	60	75.70 ± 0.46	128.3 ± 1.05
4	90	75.70 ± 0.46	128.8 ± 1.05

## Data Availability

The original contributions presented in the study are included in the article/Supplementary Materials, further inquiries can be directed to the corresponding authors.

## Supplementary Materials

The following supporting information can be downloaded at: https://www.mdpi.com/article/10.3390/jfb17060290/s1, Supplementary Table S1: The 2-factor Central Composite Design (CCD) matrix showing the 13 experimental runs, coded factor levels, and observed responses, Supplementary Table S2: Regression analysis results obtained for various responses, Y1 (Particle size) and Y2 (% Entrapment Efficiency) of the liposome formulation for fitting to different models, Supplementary Table S3: ANOVA result for various responses of Liposomes, Supplementary Table S4: Optimization of formulation validation, Supplementary Table S5: In-vitro drug release study of Liposomal Gel, Supplementary Table S6: MIC and MFC value of AN extract, IBC drug (reference), AN-AgNPs & Liposomes on *C. albicans* & *C. glabrata*, Supplementary Table S7: Effect on entrapment efficiency and particle size for liposomal dispersion from liposomal formulation during stability, Supplementary Table S8: FTIR spectral interpretation of AN Extract and AN-AgNPs; Supplementary Figure S1: MIC concentration (μg/ml) of (A) AN extract (125 ± 5.2), (B) IBC drug (Control) (8 ± 1.5), (C) AN-AgNPs (62.5 ± 4.7), (D) Liposomes on *C. albicans* (<1 ± 0.8).; Supplementary Figure S2: MIC concentration (μg/ml) of (A) AN extract (250 ± 6), (B) IBC drug (Control) (8 ± 1.5), (C) AN-AgNPs (31.25 ± 3), (D) Liposomes on *C. glabrata* (8 ± 1.5).; 1. Minimum Inhibitory Concentration (MIC); 2. Minimum Fungicidal Concentration (MFC).

## Abbreviations

The following abbreviations are used in this manuscript:

VVC	Vulvovaginal Candidiasis
EPR	Enhanced Permeability and Retention
AgNPs	Silver Nanoparticles
AN-AgNPs	*Ascophyllum nodosum*-derived Silver Nanoparticles
IBC	Ibrexafungerp citrate
QbD	Quality by Design
HSPC	Hydrogenated Soy Phosphatidyl Choline
UV-Vis	Ultraviolet-Visible
FTIR	Fourier Transform Infrared
TEM	Transmission Electron Microscopy
SEM-EDX	Scanning Electron Microscopy-Energy Dispersive X-ray
DSC	Differential Scanning Calorimetry
XRD	X-ray Diffraction
SPR	Surface Plasmon Resonance
PDI	Poly Dispersity Index
DOE	Design of Experiments
MIC	Minimum Inhibitory Concentration
MFC	Minimum Fungicidal Concentration
ANOVA	Analysis of Variance
EE	Entrapment Efficiency
O-H	Hydroxyl group
N-H	Amino group
C-H	Carbon-hydrogen bond
C=O	Carbonyl group
C-O	Carbon–oxygen bond
AgNO_3_	Silver nitrate
NaOH	Sodium hydroxide
TEA	Triethanolamine
