# Supplementary material for: Synergistic Liposomal Delivery of Ibrexafungerp Citrate and Marine-Sourced Silver Nanoparticles for Effective Management of Vulvovaginal Candidiasis
Source: J Funct Biomater. 2026 Jun 9;17(6):290. doi: 10.3390/jfb17060290 (PMC13302633; doi:10.3390/jfb17060290)
Supplement: Supplementary file 1 [file jfb-17-00290-s001.zip › jfb-4265615-supplementary.pdf]

Supplementary Information

Synergistic Liposomal Delivery of Ibrexafungerp Citrate and Marine-Sourced Silver Nanoparticles for Effective Management of Vulvovaginal Candidiasis

Pottabathula Shyam Sundar [1], Uday Kumar S. Patil [2], Thombre Pooja Sarjerao [3], Somnath D. Bhinge [4], Sunil T. Galatage [5,*], Unnam Sambamoorthy [6], Rahul J. Kadam [1], Viswas Raja Solomon [7,8] and Arehalli S. Manjappa [5,*]

1 Department of Pharmaceutical Chemistry, Vasantidevi Patil Institute of Pharmacy, Kodoli, Panahala, Kolhapur 416114, Maharashtra, India; shyam.pottabathula@yspm.in (P.S.S.); rahul.kadam@yspm.in (R.J.K.)

2 Department of Pharmaceutics, Bharati Vidyapeeth College of Pharmacy, Kolhapur 416013, Maharashtra, India; udaykumar.patil@bharatividyapeeth.edu

3 Department of Pharmaceutical Quality Assurance, Vasantidevi Patil Institute of Pharmacy, Kodoli, Panahala, Kolhapur 416114, Maharashtra, India; poojathombre2001@gmail.com.

4 Krishna Institute of Pharmacy, Krishna Vishwa Vidyapeeth (Deemed To Be University), Karad 415539, Maharashtra, India; somu1245@gmail.com

5 Department of Pharmaceutics, Vasantidevi Patil Institute of Pharmacy, Kodoli, Panhala, Kolhapur 416114, Maharashtra, India

6 Department of Pharmaceutics, Sree Dattha Institute of Pharmacy, Sheriguda, Ibrahimpatnam, Greater Hyderabad 501510, Telangana, India; unnammoorthy@sreedattha.ac.in

7 Medicinal Chemistry Research Laboratory, MNR College of Pharmacy, Sangareddy 502294, Telangana, India; vrajasolomon@gmail.com

8 Department of Chemistry, University of Saskatchewan, Saskatoon, SK S7N 5A2, Canada

* Correspondence: gsunil201288@gmail.com (S.T.G.); manju_as82@yahoo.co.in (A.S.M.)

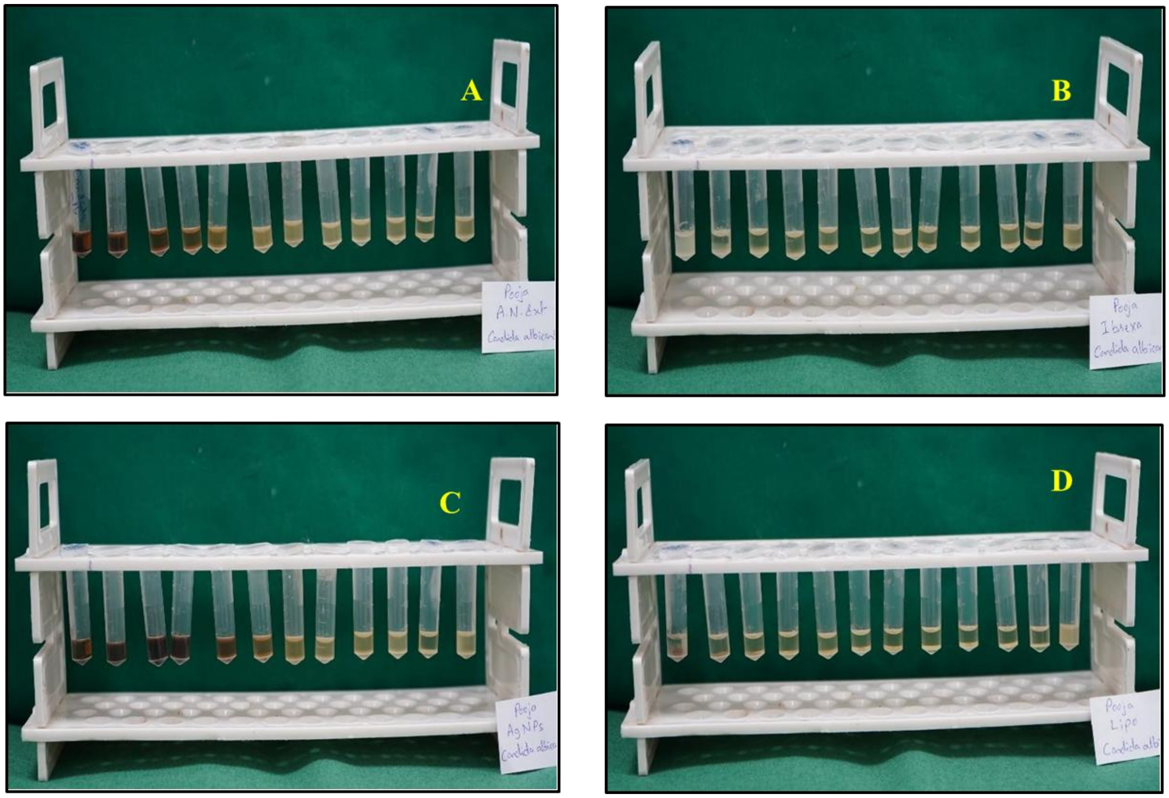

Figure S1. MIC concentration (µg/ml) of A) AN extract (125 ± 5.2), B) IBC drug (Control) (8 ± 1.5), C) AN-AgNPs (62.5 ± 4.7), D) Liposomes on *C. Albicans* (<1 ± 0.8).

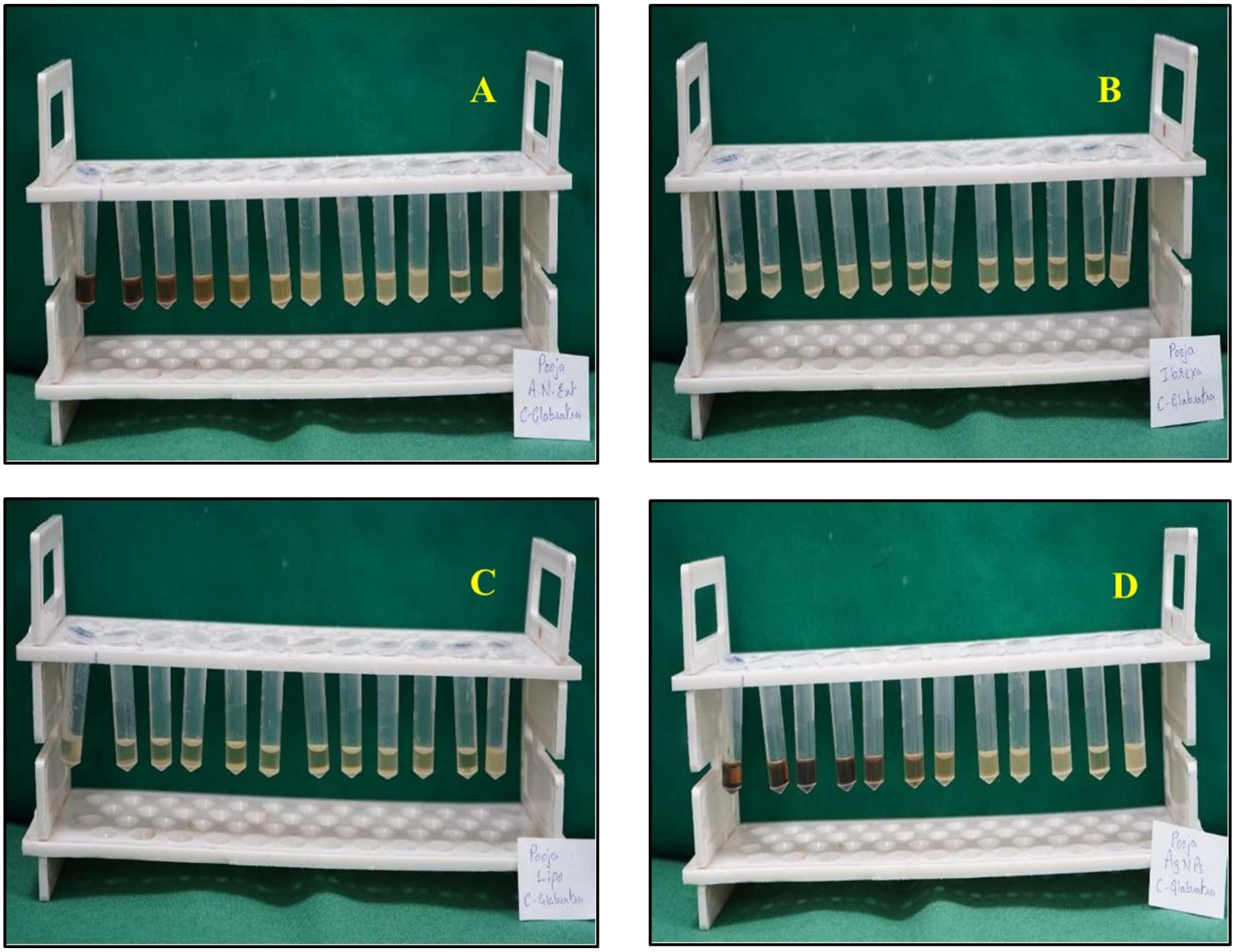

Figure S2. MIC concentration (µg/ml) of A) AN extract (250 ± 6), B) IBC drug (Control) (8 ± 1.5), C) AN-AgNPs (31.25 ± 3), D) Liposomes on *C. Glabrata* (8 ± 1.5).

Tables

Table S1. The 2-factor Central Composite Design (CCD) matrix showing the 13 experimental runs, coded factor levels, and observed responses

Batch No	X1 HSPC (mg)	X2 Cholesterol (mg)	Y1 Particle Size (nm)	Y2 % EE
L1	0	0	159.32	59.84 ± 0.06
L2	0	0	158.34	60.29 ± 0.05
L3	0	0	160	58.32 ± 0.08
L4	+1	0	128.6	74.45 ± 0.07
L5	-1	0	130.23	73.66 ± 0.06
L6	-1	+1	121.3	75.62 ± 0.07
L7	0	0	154.94	62.52 ± 0.05
L8	0	+1	156.23	70.26 ± 0.07
L9	0	-1	138.63	71.25 ± 0.08
L10	+1	+1	143.6	55.64 ± 0.06
L11	+1	+1	127.2	76.77 ± 0.07
L12	0	0	155.32	61.12 ± 0.05
L13	-1	-1	148.8	69.84 ± 0.08
**Factors**	**Coded level and actual value**			
**Independent variables**	**Low (-1)**	**Medium (0)**	**High (+1)**	
**Factor X1: Amount of HSPC (mg)**	**10**	**20**	**30**	
**Factor X2: Amount of Cholesterol (mg)**	**5**	**10**	**15**	
**Dependent Variables or Responses**	**Constraint**			
**Response Y1: Particle size (nm)**	**Minimize**			
**Response Y2: Entrapment Efficiency (%)**	**Maximize**			

Note: The design matrix consists of 13 experimental runs generated by the Central Composite Design (CCD). X1: Factor 1, HSPC (mg); X2: Factor 2, Cholesterol (mg). Y1: Response 1, Particle Size (nm); Y2: Response 2, % EE. Coded levels represent: -1 (Low), 0 (Medium), and +1 (High).

Table S2. Regression analysis results obtained for various responses Y1 (Particle size) and Y2 (% Entrapment Efficiency) of Liposome formulation for fitting to different model.

Models	SD	R^2	Adjusted R^2	Predicted R^2	PRESS	Remark
Response (Y1)						
Linear	15.24	0.0100	0.1879	0.7898	4200.50	
2FI	16.04	0.0128	0.3162	1.7957	6561.15	
**Quadratic**	**9.01**	**0.7577**	**0.5846**	**0.6720**	**3924.11**	**Suggested**
Cubic	3.62	0.9720	0.9328	0.9328	2858.17	Aliased
Response (Y2)						

Linear	7.50	0.1499	0.0202	0.5341	1014.87	
2FI	7.48	0.2389	0.0148	0.7932	1186.27	
**Quadratic**	**4.66**	**0.7699**	**0.6056**	**0.5549**	**1028.64**	**Suggested**
Cubic	2.32	0.9593	0.9023	0.6924	1119.60	Aliased

Table S3. ANOVA result for various responses of Liposomes

Source	Responses					
	Y1 (particle size)			Y2 (% Entrapment efficiency)		
	F-value	P-value Prob > F	Adequacy Precision	F-value	P-value Prob > F	Adequacy Precision
**Model**	**4.38**	**0.0398**	**5.1109**	**4.69**	**0.0338**	**5.0676**
**X1**	**0.0297**	**0.8680**		**0.8186**	**0.3956**	
**X2**	**0.2604**	**0.6255**		**3.74**	**0.0943**	
**X1X2**	**0.0800**	**0.7854**		**2.71**	**0.1438**	
**$X1^2$**	**19.94**	**0.0029**		**11.84**	**0.0108**	
**$X2^2$**	**3.35**	**0.1100**		**6.29**	**0.0405**	

Table S4. Optimization of formulation validation

**Response**	**Predicted value**	**Observed value**	% Error
Particle size	136.27	127.2	-6.65
Entrapment Efficiency	76.47	76.77	+0.39

Table S5. *In-vitro* drug release study of Liposomal Gel

**Sr.no**	**Time (hrs.)**	**% Drug release of** Pure IBC (API)	**% Drug release of IBC**	**% Drug release of AN**
**1**	0	0 ± 0	0 ± 0	0 ± 0
**2**	0.5	0.7 ± 0.05	1.7 ± 1.02	0.02 ± 0
**3**	1	1.2 ± 1.1	3.3 ± 2.15	0.5 ± 0.02

**4**	2	4.13 ± 2.01	4.48 ± 1.96	1.73 ± 0.9
**5**	4	11.48 ± 2.45	8.53 ± 1.89	5.89 ± 1.52
**6**	6	24.4 ± 2.04	15 ± 3.01	12.18 ± 1.852
**7**	8	24.4 ± 1.94	26.89 ± 3.29	23.52 ± 2.032
**8**	10	24.44 ± 1.89	44.31 ± 2.45	38.07 ± 1.45
**9**	12	24.54 ± 1.87	56.8 ± 2.01	51.3 ± 1.951
**10**	24	N/A*	72.6 ± 3.02	69.18 ± 1.753

*N/A: Not Applicable

Table S6. MIC and MFC value of AN extract, IBC drug (reference), AN-AgNPs & Liposomes on C. *Albicans* & C. *Glabrata*

Sr. No.	Test Material	MIC (µg/ml)	MFC (µg/ml)
	C. Albicans		
1	AN extract	125 ± 5.2	250 ± 6
2	IBC	8 ± 1.5	16.12 ± 2.8
3	AN-AgNPs	62.5 ± 4.7	125 ± 4
4	Liposomes	<1 ± 0.8	<1 ± 0.8
	C. Glabrata		
1	AN extract	250 ± 6	>500 ± 8
2	IBC	8 ± 1.5	16.12 ± 2.8
3	AN-AgNPs	31.25 ± 3	62.5 ± 4.7
4	Liposomes	8 ± 1.5	8 ± 1.5

Table S7. Effect on entrapment efficiency and particle size for liposomal dispersion from liposomal formulation during stability

Sr.no	No. of Days	Entrapment Efficiency (%)	Particle size (nm)

		4-8^{0}C	4-8^{0}C
1	0	76.77 ± 0.53	127.2 ± 1.23
2	30	76.12 ± 0.37	127.89 ± 1.36
3	60	75.70 ± 0.46	128.3 ± 1.05
4	90	75.70 ± 0.46	128.8 ± 1.05

Table S8. FTIR spectral interpretation of AN Extract and AN-AgNPs

**AN Extract (Wavenumber (cm^{-1}))**	**AN-AgNPs (Wavenumber (cm^{-1}))**	**Functional Group**	**Chemical Assignment**	**Role**
3893.41–3786.77	—	Free –OH stretching	Alcohols/phenols (weak/isolated OH)	Minor contribution
3429.98	3332.31	–OH / H-bonded OH	Polyphenols (phlorotannins)	Primary reduction + capping
2919.31, 2853.72	—	–CH stretching	Aliphatic chains	Structural
2732–2507	—	O–H (acid) / overtone region	Carboxylic acids	Minor
2383.35	—	CO_2 adsorption	Atmospheric CO_2	Artifact
2182–1894	—	Overtone/combination bands	Weak transitions	Negligible
1803.63	—	C=O (esters)	Lipids/esters	Minor
1595.11	1636.87	C=O stretching	Carbonyl (proteins/phenolics)	Reduction + coordination
1408.75	—	O–H bending	Phenolics/alcohols	Stabilization
1217.57	—	C–O–C stretching	Polysaccharides	Capping
1110.56, 1080.29	1015.56	C–O stretching	Polysaccharides (fucoidans)	Stabilization
867.86–707.80	918.50, 812.91	C–H bending	Aromatic/alkenes	Structural

***In-vitro* Antifungal Activity**

1. Minimum Inhibitory Concentration (MIC)

The antifungal efficacy of the AN extract, IBC drug (reference), AN-AgNPs, and the liposomal formulation was assessed against two pathogenic Candida species: *Candida albicans* and *Candida glabrata*. The MIC was determined by serial dilution in Fluid Thioglycollate Medium. An initial stock solution was prepared by combining 20 μL of the test formulation with 380 μL of sterile thioglycollate broth. A dilution series was prepared using nine additional tubes, each pre-filled with 200 μL of sterile broth. A serial dilution was performed by transferring 200 μL from the initial stock tube into the first dilution tube. Subsequently, 200 μL was transferred sequentially from tube to tube to achieve a geometric dilution series, culminating in the ninth tube. In parallel, inoculum suspensions of *C. albicans* and *C. glabrata* were prepared by transferring 5 μL of the respective stock cultures into 2 mL of thioglycollate broth. From these standardised suspensions, 200 μL of inoculum was added to each tube containing the test formulations (AN extract, IBC, AN-AgNPs, and Liposomes). The tubes were incubated anaerobically at 37 °C for 48 to 72 hours. After incubation, the tubes were visually examined for turbidity, indicating fungal growth. The MIC was defined as the lowest concentration of the formulation that completely inhibited visible growth of the Candida species.

2. Minimum Fungicidal Concentration (MFC)

The MFC assay was performed to determine whether the test samples exhibited fungistatic or fungicidal activity against two pathogenic Candida species: Candida albicans and Candida glabrata. First, agar plates were prepared from samples (AN extract, IBC drug (reference), AN-AgNPs, Liposomes) taken from MIC tubes showing microbial sensitivity, and incubated for 24–48 hours. The next day, colony counts were performed to assess fungal viability. In this context, an extract showing no colony formation on the agar was classified as fungicidal, indicating complete destruction of the organism. Conversely, any growth indicated a fungistatic effect, meaning the sample halted growth without killing the fungi.
